# Stress-induced MAPK (SIMK)-dependent organization of microtubules in alfalfa

**DOI:** 10.1186/s43897-026-00231-0

**Published:** 2026-06-09

**Authors:** Kateřina Hlaváčková, Miroslav Ovečka, Olga Šamajová, Jozef Šamaj

**Affiliations:** Department of Biotechnology, Faculty of Science, Palacký University Olomouc, Olomouc, Czech Republic

**Keywords:** *Medicago sativa*, Development, Cytoskeleton, MAPK, Protein abundance, Fluorescent molecular marker

## Abstract

Mitogen-activated protein kinases (MAPKs) modulate the organization of the plant cytoskeleton. The spatial organization of microtubules is critical for cell division, polarity, shape control, and elongation during plant growth and morphogenesis. Here, we analyzed the impact of the stress-induced MAPK (SIMK) abundance on the tubulin cytoskeleton in different plant organs and tissues of an important legume crop species, alfalfa (*Medicago sativa* L.). For this purpose, we have established unique transgenic double lines with genetically manipulated SIMK, possessing microtubular molecular fluorescent marker, the tag RED FLUORESCENT PROTEIN-TUBULIN ALPHA 6 (tagRFP-TUA6). We show that lower or higher SIMK abundance can change cell division planes (CDPs) and phragmoplast orientations in roots. In addition, the transgenic line with downregulated *SIMK* shows disordered and disorganized microtubules, and a reduced degree of microtubule bundling mainly in leaves and stems. This may be linked to smaller habitus, shorter stems, and smaller leaves. Obtained results indicate that the genetic manipulation of SIMK abundance has effect on microtubule organization and plant development in alfalfa. This study also paves the way for testing anti-microtubular drugs on alfalfa and for biotechnological use of the newly-developed lines.

**Supplementary Information:**

The online version contains supplementary material available at 10.1186/s43897-026-00231-0.

## Core

SIMK abundance manipulated by genetic engineering affects the organization of microtubules in roots, stems, and leaves of an important legume crop, alfalfa (*Medicago sativa* L.). Microtubule inhibitors are used against weeds limiting alfalfa yield, but might have undesirable effects also on alfalfa itself. Therefore, we developed unique alfalfa transgenic lines for in vivo analysis of microtubules as a useful tool for future testing of various microtubule inhibitors directly at the molecular level.

## Gene & accession numbers

Information about the genes in this article is available at The National Center for Biotechnology (https://www.ncbi.nlm.nih.gov/) with the following accession numbers: *MsSIMK* (X66469) and *AtTUA6* (At4G14960).

## Introduction

Actin filaments, microtubules, and associated proteins are essential components of plant cytoskeleton, roles of which are multifaceted as it participates in a wide range of cellular processes including cellular transport, communication, signaling or cell division, expansion, and differentiation (Brown and Lemmon 2001; Kost and Chua 2002; Sheahan et al. 2004; Breuer et al. 2017; Vavrdová et al. 2019b; Motta and Schnittger 2021). Microtubules are tubulin polymers capable of rapid elongation and shrinkage, which is known as dynamic instability (Elliott and Shaw 2018). This, together with other mechanisms such as nucleation, severing, branching, and bundling, determines the spatiotemporal organization of microtubules, which is crucial for plant morphogenesis, development, and growth (Goddard et al. 1994; Wasteneys and Ambrose 2009; Sedbrook and Kaloriti 2008; Li et al. 2015; Elliott and Shaw 2018). Microtubule organization and dynamic behavior are mainly controlled by microtubule-associated proteins (MAPs), kinesins, plus-end binding (EB1) proteins, γ-tubulin, severing protein katanin, microtubule-destabilizing protein 25 (MDP25), phospholipase Dα1, and others (Hamada 2007, 2014; Gardiner 2013; Šamajová et al. 2013; Krtková et al. 2016; Vavrdová et al. 2019b). Some of these proteins might be regulated by signaling molecules, including mitogen-activated protein kinases (MAPKs) (Beck et al. 2010; Müller et al. 2010; Zhang et al. 2016; Jiang et al. 2022), small GTPases called Rho of plants (ROPs), calcium, and phosphatidic acid (Komatsu et al. 2007; Yalovsky et al. 2008; Zhang et al. 2012), thus coupling microtubules to the external conditions and mediating their developmental and conditional reorganization.

Plant MAPKs constitute a network of signaling cascades functioning downstream of receptors and transducing the external stimuli to coordinate cellular responses and shape the plant body in its adaptation to the ever-changing environment through protein (de)-phosphorylation (Widmann et al. 1999; Ichimura et al. 2002; Ma and Nicolet 2023). Activated MAPKs phosphorylate various downstream targets, including transcription factors, enzymes, cytoskeletal proteins, or other kinases, and regulate a broad range of cellular and developmental processes (Rasmussen et al. 2012; Komis et al. 2018; Sun and Zhang 2022). Considering that a single MAPK can be regulated by more than one MAPKKK/MAPKK pair, these signaling modules represent versatile tools in the regulation of microtubule organization. So far, direct phosphorylation of tubulins by MAPKs has not been shown. Still, there are few examples demonstrating the functional modulation of tubulin cytoskeleton organization and dynamics by MAPKs in plants. In Arabidopsis, NUCLEUS- AND PHRAGMOPLAST-LOCALIZED KINASE 2 and 3 (ANP2 and ANP3), MPK4, and proteins from the MAP 65-kDa (MAP65) family are essential for the proper organization of cortical microtubules in epidermal cells, microtubule-dependent cell growth mechanisms, and cytokinesis. Both *anp2anp3* and *mpk4* mutants show mitotic and cytokinetic defects, root and root hairs malformations, and heavily bundled microtubules (Beck et al. 2010, 2011). Co-sedimentation and co-immunoprecipitation showed physical association of AtMAP65-1 with MPK4 and MPK6 and its phosphorylation by both MAPKs (Smertenko et al. 2006; Beck et al. 2010). MPK4 localizes to the phragmoplast, but when fused to GREEN FLUORESCENT PROTEIN (GFP) or YELLOW FLUORESCENT PROTEIN (YFP), the signal concentrates rather in the phragmoplast midzone and developing cell plate (Kosetsu et al. 2010; Beck et al. 2011). MPK6 associates with mitotic microtubules in root meristematic cells (Müller et al. 2010; Smékalová et al. 2014; Kohoutová et al. 2015), likely in an activation-dependent manner (Smékalová et al. 2014), as well as with cortical microtubules in an activated state (Vavrdová et al. 2019a). Cytoskeletal targets of MPK6 include MAP65-1 (Smékalová et al. 2014) and EB1c (Kohoutová et al. 2015), but MPK6 interestingly interacts with γ-tubulin, anticipating its role in the microtubule nucleation process (Kohoutová et al. 2015). The best-studied MAPK module implicated in cytoskeletal regulation is the NACK-PQR pathway in tobacco (*Nicotiana tabacum*) (Takahashi et al. 2004), composed of NPK1 (MAPKKK) (Nishihama et al. 2001), the NQK1(MAPKK) (Soyano et al. 2003; Takahashi et al. 2004), and the NRK1 (MAPK) (Nishihama et al. 2001). NRK1 phosphorylates MAP65-1, the major microtubule-crosslinking MAP, thus controlling the rate of centrifugal phragmoplast expansion (Sasabe and Machida 2006). In legume crop alfalfa (*Medicago sativa* L.), one of the first MAPKs found to be involved in cytoskeleton regulation was Medicago MAPK2 (MMK2) (Jonak et al. 1995), targeting a 39 kDa protein in the detergent-resistant cytoskeleton of carrot cells (Jonak et al. 1995). Another alfalfa MAPK, MMK3, is highly abundant and active in young organs, present during all stages of the cell cycle, and becomes activated in a microtubule-dependent manner (Bögre et al. 1999). The stress-induced MAPK (SIMK), an alfalfa orthologue of AtMPK6, is known to be involved in alfalfa − *S. meliloti* symbiotic interaction (Hrbáčková et al. 2021; Hlaváčková et al. 2023), shoot biomass production (Hrbáčková et al. 2021), resistance to oxidative stress (Sojka et al. 2024), and together with the dynamic actin cytoskeleton also in the regulation of alfalfa root hair tip growth (Šamaj et al. 2002). Although SIMK colocalization with mitotic microtubules in alfalfa root meristematic cells, which was enhanced by an osmotic stress, has been observed (Baluška et al. 2000) and SIMK colocalized with mitotic microtubules after their stabilization by taxol (Šamaj et al. 2004), its interplay with microtubules in alfalfa remains poorly characterized.

Here, we studied the impact of SIMK abundance on the tubulin cytoskeleton using newly developed alfalfa transgenic double lines with genetically manipulated SIMK co-expressing a fluorescent molecular marker for microtubules. We provide a comprehensive overview of microtubule organization in different organs and tissues of alfalfa depending on SIMK genetic manipulation, along with detailed biochemical and phenotypic analysis. The obtained results showed disordered and disorganized microtubules, shifted cell division planes (CDPs) and phragmoplasts, helical growth of secondary leaf veins, and impaired phenotypes of the above-ground part in the SIMKKi^tagRFP−TUA6^ line (causing RNAi-based *SIMK* strong downregulation). This suggests that SIMK abundance may have a regulatory effect on microtubule organization, cell morphogenesis, CDPs orientation, and plant development in alfalfa. Considering the biotechnological potential of alfalfa and the central role of the cytoskeleton in plant growth and development, addressing the detailed organization of the tubulin cytoskeleton in legume crops is highly desirable. Its detailed characterization will contribute to a better understanding of kinase-dependent cytoskeletal rearrangements in relation to plant growth, morphogenesis, and development, and might help to improve agronomically important traits. Moreover, alfalfa is a legume cover crop that suffers, as other widely utilized horticulture crops, from ecological and competitive pressure of weeds. Among plethora of different protecting chemicals, microtubule inhibitors represent an important class of herbicides in weed management (Paul et al. 2024; Torra and Alcántara-de la Cruz 2022). These chemicals disrupt the microtubule network, which cause direct inhibition of cell division and growth in plant cells (Chen et al. 2021) as an integrated approach in weed management. New microtubule inhibitors should have improved selectivity, minimal toxicity to crops, and reduced environmental impact, together with the aim to circumvent acquired resistance in weeds. To test and visualize molecular mechanism of their effects on microtubules *in planta*, we need new alfalfa lines containing molecular microtubule markers. Therefore, to develop biological material visualizing microtubules in vivo, as a potent tool for future studies on alfalfa weed management and biotechnological improvements, is desirable.

## Results

### Co-visualization of microtubules with GFP-tagged SIMK

**Fig. 1:**
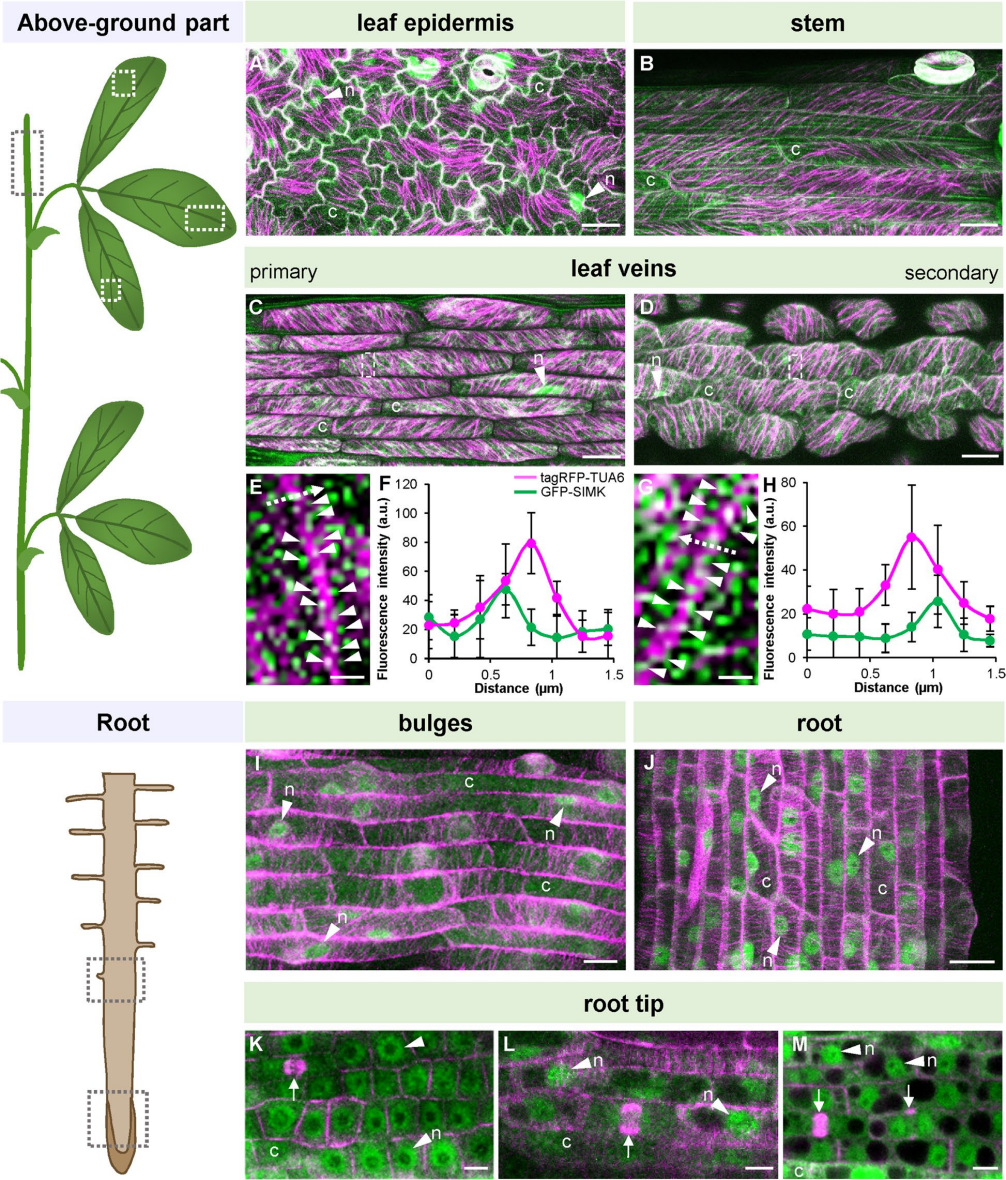
Co-visualization of microtubules with GFP-tagged SIMK in the alfalfa GFP-SIMK^tagRFP−TUA6^ overexpression line. Representative images showing organization of cortical microtubules in leaf epidermal cells **(A)**, stem epidermal cells (**B**), primary (**C**) and secondary (**D**) leaf veins, root hair bulges (**I**) and mature part of the root (**J**) or mitotic microtubules in root tips (**K-M**) co-visualized with GFP-tagged SIMK in the transgenic double GFP-SIMK^tagRFP−TUA6^ overexpression line. Representative detailed images of GFP-SIMK association with cortical microtubules depicted in the white dashed boxes in C-D are shown in **E**, **G**. Fluorescence intensity profiles of GFP-SIMK (green) and tagRFP-TUA6 (magenta) distribution in primary (**F**; *N* = 6) and secondary (**H**; *N* = 7) leaf vein cells along the measured arrow shown in (**E, G**). Position of cell types in different plant organs imaged by confocal laser scanning microscope in (A-I) are schematically shown in the drawings on the left and highlighted by white or grey dashed boxes. Note mitotic microtubules in mitotic spindles (**K, L**) or early and late phragmoplasts (**M**) as shown by white arrows. Microtubules are pseudocolored in magenta; n = nucleus, c = cytoplasm. Scale bar = 20 μm (A-D; I-J), 1 µm (E, G), and 10 µm (K-M)

In this work, we have developed two alfalfa transgenic double lines with genetically manipulated SIMK abundance (SIMKKi^tagRFP−TUA6^ downregulating *SIMK* and GFP-SIMK^tagRFP−TUA6^ upregulating *SIMK*) and simultaneously co-expressing a tubulin cytoskeleton marker, tagRFP-TUA6, through stable transformation of alfalfa leaf explants. Detailed co-visualization of cortical and mitotic microtubules with GFP-tagged SIMK was documented by the confocal laser scanning microscope in cells of different alfalfa organs and tissues of the above-ground part (Fig. 1A-D) and root (Fig. 1I-M) of the transgenic double GFP-SIMK^tagRFP−TUA6^ overexpression line, including leaf epidermis (Fig. 1A), stem epidermal cells (Fig. 1B), primary (Fig. 1C) and secondary (Fig. 1D) leaf veins, bulges of emerging root hairs (Fig. 1I), mature part of the root (Fig. 1J), and the root tip (Fig. 1K-M). Subcellular localization of GFP-SIMK was in all cell types of both the above-ground part and the root (Fig. 1) predominantly nuclear and cytoplasmic. Interestingly, detailed analysis from representative profiling of fluorescence intensity distribution along well-arranged cortical microtubules in cells of primary (Fig. 1C) and secondary (Fig. 1D) leaf veins showed a close association of spot-like distributed GFP-SIMK with cortical microtubules (Fig. 1E-F; G-H). Cortical microtubules were also observed in bulges of emerging root hairs and mitotic microtubules in dividing cells of root meristems. In trichoblasts with root hair bulges were microtubules aligned obliquely to the cell axis, looping through the tip, in which GFP-SIMK was also present (Fig. 1I). The tagRFP-TUA6 marker delineated also mitotic microtubules in dividing cells of root tips as revealed by visualization of mitotic spindles (Fig. 1K-L; white arrows), and early or late phragmoplasts (Fig. 1M; white arrows). In dividing cells, subcellular localization of GFP-SIMK was also observed in phragmoplasts (Fig. 1M), which was independently confirmed by immunolabeling (Suppl. Fig. S1G-I).

### SIMK-dependent cortical microtubule organization

**Fig. 2:**
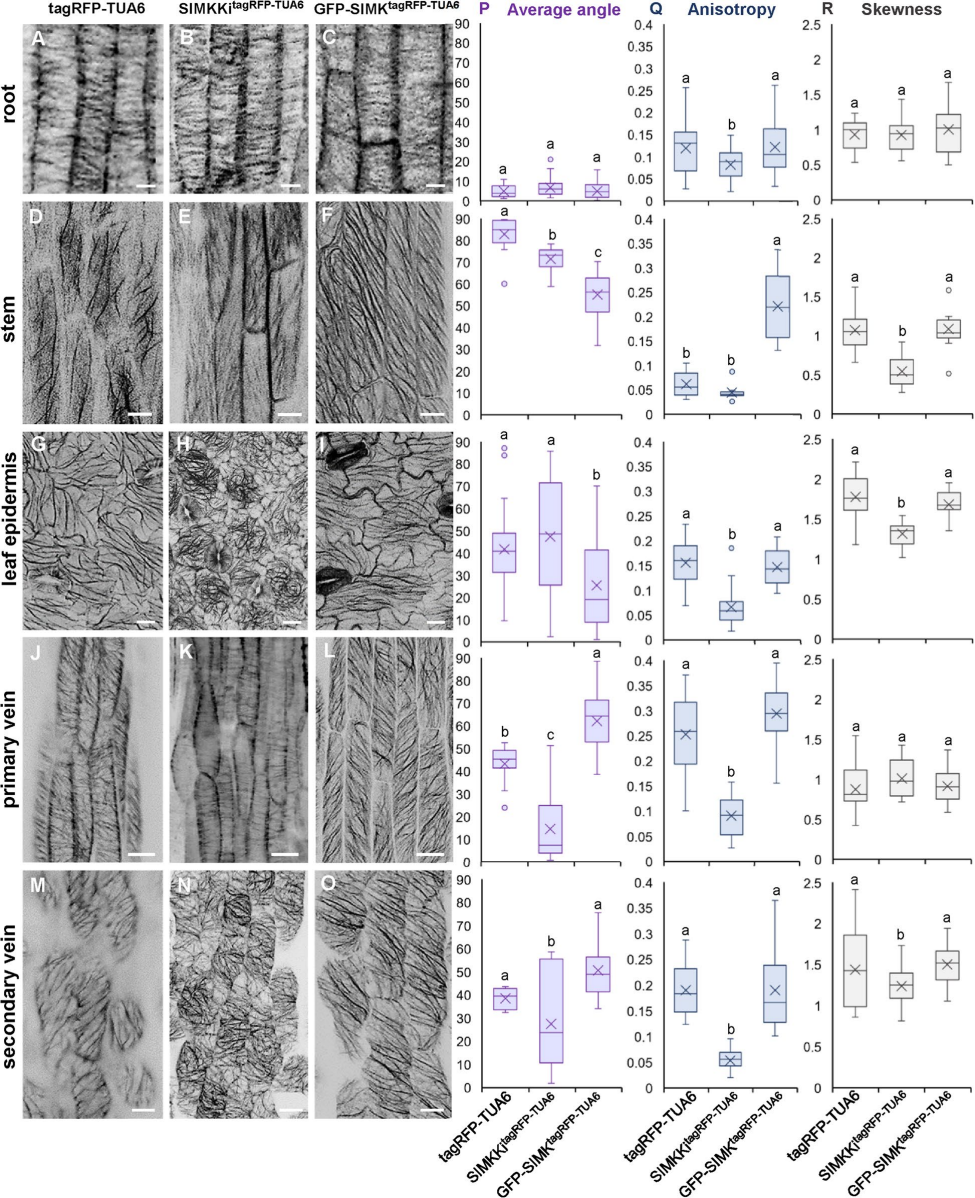
Structural organization of cortical microtubules visualized by tagRFP-TUA6 cytoskeletal marker in alfalfa transgenic lines. Representative images of cortical microtubule organization in cells of root tips (**A-C**), stem epidermis (**D-F**), leaf epidermis (**G-I**), primary (**J-L**) and secondary (**M–O**) leaf veins of the transgenic tagRFP-TUA6 (**A, D, G, J, M**), SIMKKi^tagRFP−TUA6^ (**B, E, H, K, N**), and GFP-SIMK^tagRFP−TUA6^ (**C, F, I, L, O**) lines. Quantification of microtubule average angle of orientation (**P**), anisotropy (**Q**), and skewness (**R**) in root tip cells (*N* = 24 for tagRFP-TUA6; *N* = 36 for SIMKKi^tagRFP−TUA6^; *N* = 20 for GFP-SIMK^tagRFP−TUA6^), stem epidermal cells (*N* = 25; *N* = 20; *N* = 29), leaf epidermal cells (*N* = 19; *N* = 19; *N* = 24), and primary (*N* = 19; *N* = 20; *N* = 21), and secondary (*N* = 26; *N* = 33; *N* = 26) leaf vein cells using the Fibril Tool plug-in of the ImageJ software. Lowercase letters indicate statistical significance between lines according to one-way ANOVA with post-hoc Tukey HSD test (*P* < 0.05). Scale bar = 10 μm (A-O)

These stably transformed alfalfa transgenic double lines provided a unique tool and opportunity to characterize how SIMK abundance correlates with the organization of cortical and mitotic microtubules in cells of different tissues and organs of intact plants. To quantitatively determine differences in cortical microtubule organization, a detailed semi-quantitative microscopic analysis was performed. Organization of cortical microtubules in the transgenic double lines SIMKKi^tagRFP−TUA6^ and GFP-SIMK^tagRFP−TUA6^ and in the transgenic tagRFP-TUA6 line was characterized in root tips (Fig. 2A-C), stem epidermal cells (Fig. 2D-F), leaf epidermis (Fig. 2G-I), and in leaf primary (Fig. 2J-L) and secondary (Fig. 2M-O) veins. The tubulin cytoskeleton was then quantified by measuring the average angle of cortical microtubule orientation (Fig. 2P), the degree of anisotropy within cortical array showing how well microtubules are ordered (Fig. 2Q), and skewness determining the extent of microtubule bundling (Fig. 2R). Cortical microtubule organization differed depending on the imaged plant organ and tissue, and comparison between the SIMKKi^tagRFP−TUA6^, GFP-SIMK^tagRFP−TUA6^, and tagRFP-TUA6 lines revealed differences depending on SIMK abundance.

In cells of root tips were cortical microtubules mostly perpendicular to the cell elongation axis (Fig. 2A-C). While there were no significant differences in average angle of cortical microtubule orientation (Fig. 2P), which was around 5°, and the degree of bundling (Fig. 2R) in root tip cells of the transgenic tagRFP-TUA6, SIMKKi^tagRFP−TUA6^, and GFP-SIMK^tagRFP−TUA6^ lines, microtubule anisotropy was significantly reduced to 0.02 ± 0.15 in the transgenic SIMKKi^tagRFP−TUA6^ line (Fig. 2Q).

In stem epidermal cells, the orientation of cortical microtubules differed among all respective lines with the average angle of 55° in the transgenic GFP-SIMK^tagRFP−TUA6^ line, 71° in the SIMKKi^tagRFP−TUA6^ line, and 83° in the tagRFP-TUA6 line (Fig. 2P). In addition, the highest anisotropy of 0.13 ± 0.34, showing well-ordered cortical microtubules, was revealed in the GFP-SIMK^tagRFP−TUA6^ line (Fig. 2Q), in which, together with the transgenic tagRFP-TUA6 line, the skewness was higher compared to the SIMKKi^tagRFP−TUA6^ line (Fig. 2R).

In leaf epidermal cells of the transgenic SIMKKi^tagRFP−TUA6^ line were cortical microtubules disorganized showing a somewhat biased array with no preferential direction, but in the transgenic tagRFP-TUA6 and GFP-SIMK^tagRFP−TUA6^ lines they were aligned in parallel bundles within the cell, running across neck regions (Fig. 2G-I). The average angle of cortical microtubule orientation was in these cells generally lower, with 42° in the transgenic tagRFP-TUA6 line, 48° in the transgenic SIMKKi^tagRFP−TUA6^ line, and only 25° in the GFP-SIMK^tagRFP−TUA6^ line (Fig. 2P). In the transgenic SIMKKi^tagRFP−TUA6^ line, cortical microtubule anisotropy was significantly reduced to 0.02 ± 0.19 (Fig. 2Q) and skewness also decreased (Fig. 2R).

Differences in cortical microtubule organization were also observed in leaf veins. In the SIMKKi^tagRFP−TUA6^ line were cortical microtubules in cells of primary leaf veins arranged transversely to the cell axis but rather obliquely with different angles in the transgenic tagRFP-TUA6 and GFP-SIMK^tagRFP−TUA6^ lines (Fig. 2J-L). The average angle of microtubule orientation in these cells was 43° and 62° for tagRFP-TUA6 and GFP-SIMK^tagRFP−TUA6^ lines, respectively, while it was only 26° for SIMKKi^tagRFP−TUA6^ line (Fig. 2P). Cells of secondary leaf veins were smaller and wider. While in the SIMKKi^tagRFP−TUA6^ line displayed their cortical microtubules random organization (Fig. 2N), in the transgenic tagRFP-TUA6 (Fig. 2M) and GFP-SIMK^tagRFP−TUA6^ (Fig. 2O) lines were, similarly to primary veins, aligned obliquely to the cell elongation axis. In the transgenic tagRFP-TUA6 and GFP-SIMK^tagRFP−TUA6^ lines were cortical microtubules in cells of secondary leaf veins arranged obliquely with average angles of 38° and 50°, whereas in the SIMKKi^tagRFP−TUA6^ line, they were aligned under an average angle of about 27° (Fig. 2P). Anisotropy was in the SIMKKi^tagRFP−TUA6^ line significantly reduced to 0.02 ± 0.16 in primary and to 0.02 ± 0.09 in secondary leaf veins (Fig. 2Q). In skewness, there were no significant differences among the respective lines in primary leaf veins but in secondary leaf veins, skewness was lower in the SIMKKi^tagRFP−TUA6^ line (Fig. 2R) indicating less bundled microtubules. Moreover, in both leaf epidermal and secondary leaf vein cells of the SIMKKi^tagRFP−TUA6^ line, microtubules were atypically branched and disorganized (Fig. 2H, N), but also right-handed helical growth of secondary leaf veins was observed (Fig. 2N). Generally, these results showed that in the transgenic SIMKKi^tagRFP−TUA6^ double line with downregulated *SIMK*, cortical microtubules were disorganized, less ordered, and less bundled, mainly in the above-ground part, which indicates a possible impact on cell morphogenesis and plant development.

**Fig. 3:**
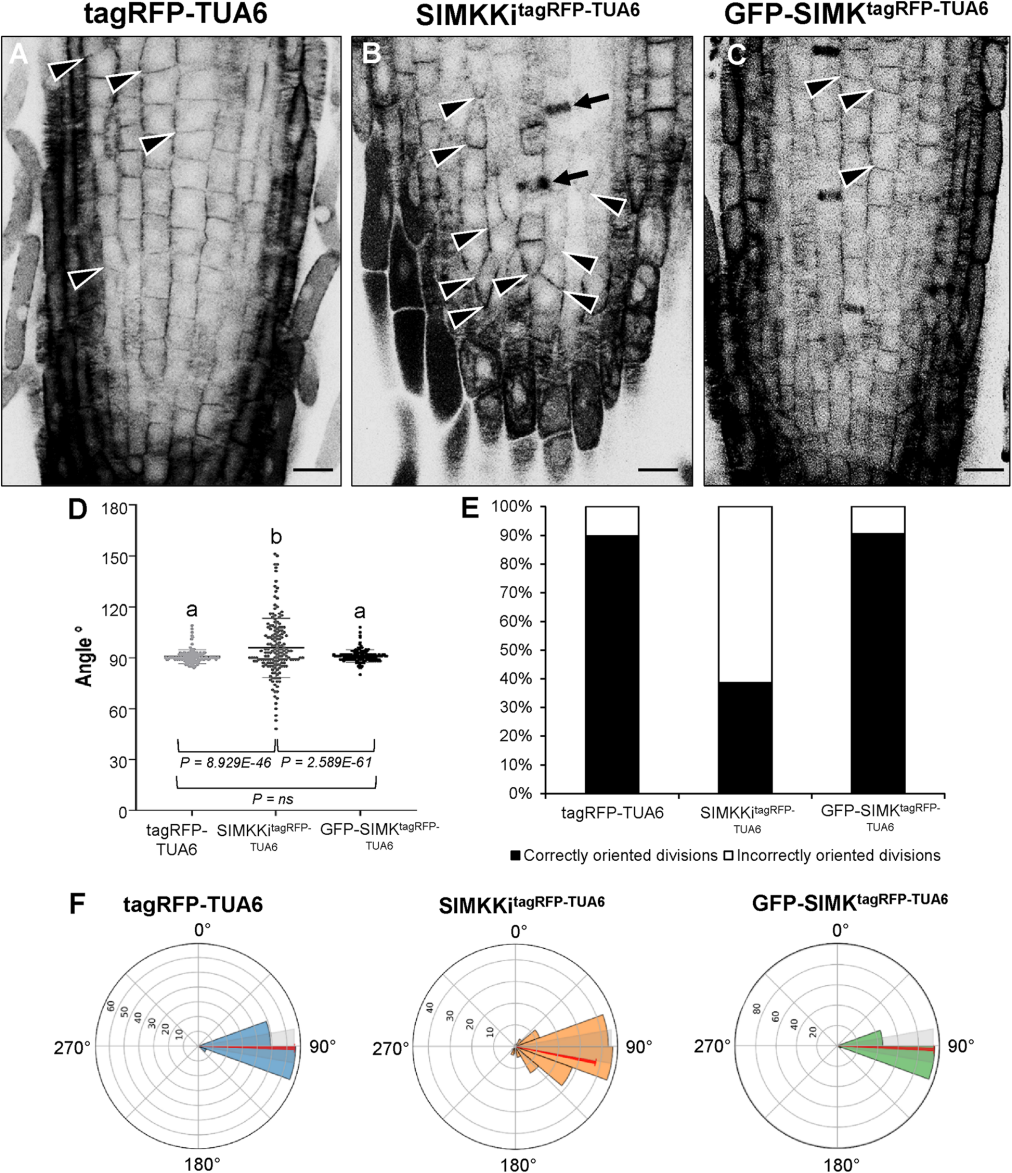
Cell division planes (CDPs) orientation in root meristems of alfalfa microtubule-reporter lines. Longitudinal optical sections comparing root tips of the transgenic tagRFP-TUA6 (**A**) and the transgenic double SIMKKi^tagRFP−TUA6^ (**B**), and GFP-SIMK^tagRFP−TUA6^ (**C**) lines. Note the abnormal pattern and the variable size of cell files caused by shifted CDPs in the transgenic double SIMKKi^tagRFP−TUA6^ line indicated by arrowheads in (**B**). Arrows in (**B**) show the shifted positioning of phragmoplasts. Quantification of cross-wall orientation between neighbouring cells, indicating the division angle in epidermal cell files (**D**) of tagRFP-TUA6 (*N* = 127), SIMKKi^tagRFP−TUA6^ (*N* = 168), and GFP-SIMK^tagRFP−TUA6^ (*N* = 138) lines. Scatter plots represent the distribution of individual division angle values and plot mean with SD. Lowercase letters indicate statistical significance in mean angle between lines according to one-way ANOVA with post-hoc Tukey HSD test (*P* < 0.05). *P*-values in (**D**) show statistical significance (*P* < 0.05) in angle dispersion between lines according to the *F*-test. Relative frequencies of angle distributions in epidermal cell files of tagRFP-TUA6 (*N* = 127), SIMKKi^tagRFP−TUA6^ (*N* = 168), and GFP-SIMK^tagRFP−TUA6^ (*N* = 138) lines (**E**), showing the percentage of correctly (90° ± 5%) and incorrectly (less than 85° and more than 95°) oriented CDPs. Rose plots of CDPs in tagRFP-TUA6, SIMKKi^tagRFP−TUA6^, and GFP-SIMK^tagRFP−TUA6^ lines (**F**). Each rose plot visualize distribution of CDP within one line, highlighting mean CDP orientation (red line). Scale bar 20 µm (A-C)

In addition, shifted CDPs, abnormal patterns, and variable sizes of cell files and aberrant positioning of phragmoplasts were evident in root epidermal cells of the SIMKKi^tagRFP−TUA6^ line compared to the transgenic tagRFP-TUA6 and GFP-SIMK^tagRFP−TUA6^ lines (Fig. 3A-C). Quantitative evaluation of cross walls orientations between neighbouring cells respective to root longitudinal axis was performed by measuring the angle between the cross and the longitudinal wall of the same cell. Such measurements revealed relatively close angle distribution between 84°˗109° in the tagRFP-TUA6 and between 80°˗108° in the GFP-SIMK^tagRFP−TUA6^ lines while it was much widely distributed between 48°˗151° in the SIMKKi^tagRFP−TUA6^ line (Fig. 3D, F). Relative frequencies of angle distributions confirmed the prevalence of transverse CDPs orientation in epidermis of the tagRFP-TUA6 and GFP-SIMK^tagRFP−TUA6^ lines (Fig. 3E). In contrast, lower number of perpendicular CDPs orientation was present in epidermal cell files of the SIMKKi^tagRFP−TUA6^ line, in which oblique CDPs orientation increased (Fig. 3E). For visualization of CDPs orientation in root epidermal cells of control RSY and transgenic SIMKKi and GFP-SIMK lines, the vital red-fluorescent styryl dye FM4-64 was used. Quantification revealed that although the differences in mean angles were not statistically significant, wider angle distribution was evident in the SIMKKi line compared to the control RSY and transgenic GFP-SIMK lines (Suppl. Fig. S2A). Relative frequencies of angle distribution then confirmed the prevalence of transversely oriented CDPs in RSY and GFP-SIMK lines and increased number of obliquely oriented CDPs in the SIMKKi line (Suppl. Fig. S2B). Most importantly, the angle dispersion in both SIMKKi^tagRFP−TUA6^ (Fig. 3D) and SIMKKi (Suppl. Fig. S2A) lines significantly differs from that in tagRFP-TUA6 and GFP-SIMK^tagRFP−TUA6^ (Fig. 3D) and also RSY and GFP-SIMK (Suppl. Fig. S2A) lines, respectively (Table S1).

Visualization of how cortical and mitotic microtubules are arranged in cells of different tissues and organs is highly desirable in intact living plants. Having this tool, along with the use of double transgenic lines with genetically-manipulated SIMK, the relationship between SIMK abundance and microtubules can be directly addressed.

### Immunolocalization of microtubules and SIMK

We have also used an alternative immunolocalization whole mount method to study microtubules in root tips of the GFP-SIMK line, as well as control RSY, and SIMKKi lines, showing contrasting abundances of SIMK (see immunoblot results in Fig. 5). Concerning cortical microtubules, well-organized arrays were observed in control RSY (Suppl. Fig. S3A) and GFP-SIMK lines (Suppl. Fig. S3C), which were more random and irregularly distributed in the SIMKKi line (Suppl. Fig. S3B). Additionally, co-immunolabeling revealed that SIMK was associated with phragmoplasts and cortical microtubules of control RSY and GFP-SIMK lines, but this was not the case for very low-abundant SIMK in the SIMKKi line (Suppl. Fig. S1, S3). SIMK also showed at least partial association with bundling/branching points at cortical microtubules in control RSY and GFP-SIMK lines, but not in the SIMKKi line (Suppl. Fig. S3). Detailed images of cortical microtubules and SIMK (Suppl. Fig. S4A-C) in GFP-SIMK line showed that, in addition to cytoplasmic presence, SIMK in the form of spots was associated also with the points of microtubules, where their bundling/branching events occurred (Suppl. Fig. S4D-L). These data indicate that microtubule organization in the studied lines likely depends on SIMK abundance.

### Biochemical characterization of alfalfa transgenic double lines

**Fig. 4:**
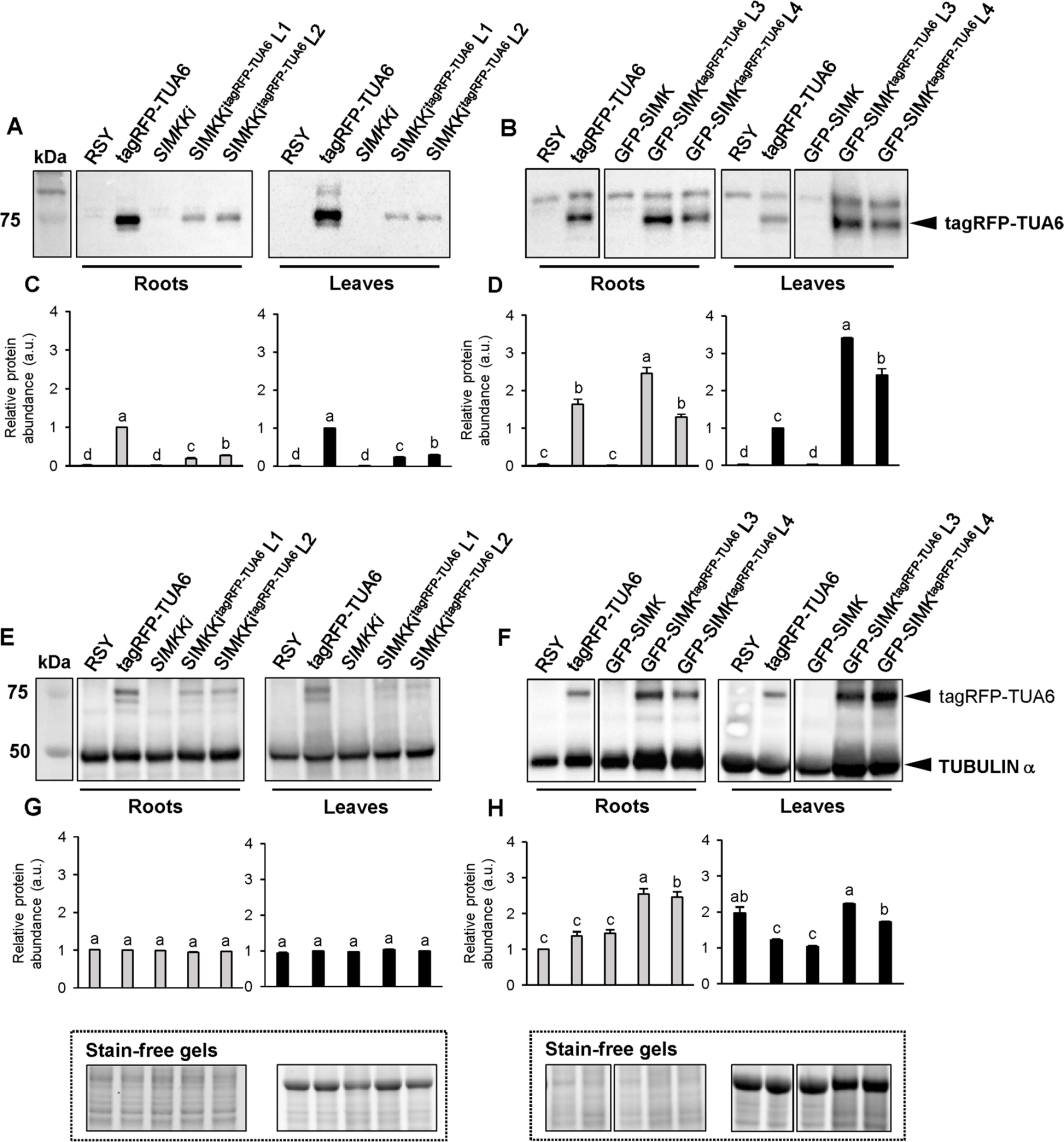
Immunoblot analysis of total tagRFP-TUA6 and endogenous TUBULIN α proteins. Western blot detection of tagRFP-TUA6 bands using the anti-RFP antibody (**A-B**) and TUBULIN α bands using the anti-TUBULIN α antibody (**E–F**) in root and leaf tissue of control RSY, transgenic tagRFP-TUA6, SIMKKi, and GFP-SIMK as well as transgenic double SIMKKi^tagRFP−TUA6^ (L1 and L2) and GFP-SIMK^tagRFP−TUA6^ (L3 and L4) lines. Arrowheads point to 76 kDa corresponding to the tagRFP-TUA6 fusion protein (**A-B; E–F**) and to 50 kDa corresponding to the endogenous TUBULIN α protein (**E–F**). Relative tagRFP-TUA6 (**C-D**) and TUBULIN α (**G-H**) protein levels in roots (grey) and leaves (black) of control, transgenic, and double transgenic plants are shown in graphs under respective PVDF membranes. Lowercase letters indicate statistical significance between lines according to one-way ANOVA with post-hoc Tukey HSD test (*P* < 0.05). Error bars show ± SD

Prepared alfalfa double lines were biochemically characterized to detect the abundance of the tagRFP-TUA6 fusion protein, TUBULIN α, and SIMK proteins using the immunoblotting method. Firstly, tagRFP-TUA6 (Fig. 4A-B) and tubulin α (Fig. 4E-F) proteins were detected in both independent lines for each stably transformed transgenic double line (SIMKKi^tagRFP−TUA6^ L1 and L2 and GFP-SIMK^tagRFP−TUA6^ L3 and L4). The abundance level of the tagRFP-TUA6 fusion protein with a molecular mass around 76 kDa was quantified (Fig. 4C-D). Relative tagRFP-TUA6 protein abundance was strongly decreased in roots of the transgenic line SIMKKi^tagRFP−TUA6^ L1 to approximately 26.99% and in L2 to approximately 19.18% and in leaves to 26.67% in L1 line and to 28.98% in L2 line to the respective control (Fig. 4C). On the other hand, in the transgenic tagRFP-TUA6 lines carrying the construct *35S::GFP:SIMK* was the relative abundance of tagRFP-TUA6 fusion protein 0.82 times higher in roots and 2.41 times higher in leaves of the L3 line and 1.41 times higher in leaves of the L4 line compared to its relative abundance in the transgenic tagRFP-TUA6 line (Fig. 4D).

In addition, TUBULIN α protein was also detected (Fig. 4E-F) and quantified (Fig. 4G-H) in all analyzed lines showing no difference in its abundance neither in roots or leaves of the transgenic SIMKKi^tagRFP−TUA6^ L1 and L2 lines (Fig. 4G) but was approximately 1.6 and 1.54 times higher in roots of the GFP-SIMK^tagRFP−TUA6^ L3 and L4 lines, respectively, and 0.27 times higher in leaves of the GFP-SIMK^tagRFP−TUA6^ L3 line (Fig. 4H).

**Fig. 5:**
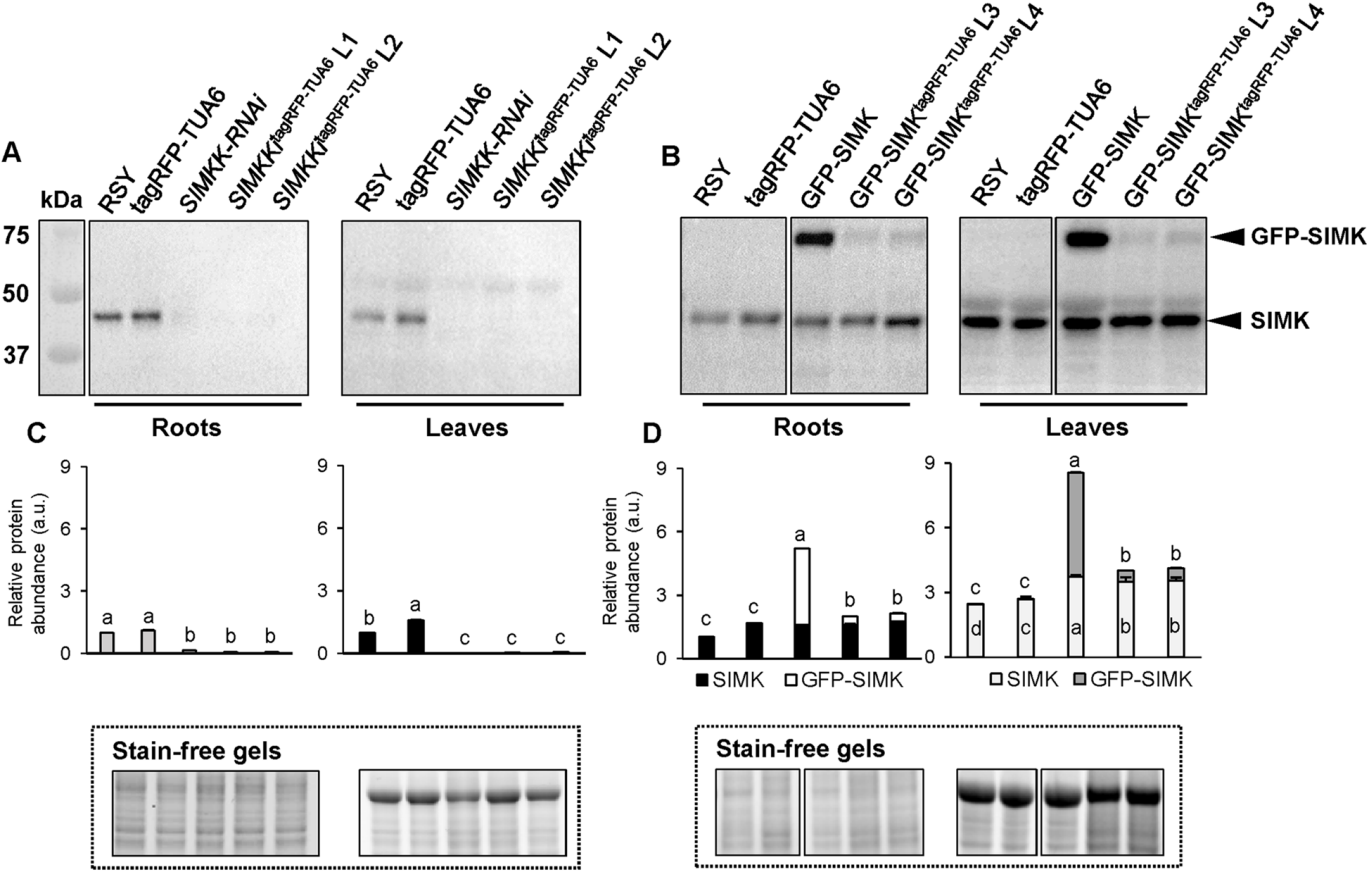
Immunoblot analysis of total endogenous SIMK and total GFP-SIMK proteins. Western blot detection of SIMK and GFP-SIMK bands using the anti-MPK6 antibody (**A-B**) in root and leaf tissue of control RSY, transgenic tagRFP-TUA6, SIMKKi, and GFP-SIMK, as well as transgenic double SIMKKi^tagRFP−TUA6^ (L1 and L2) and GFP-SIMK^tagRFP−TUA6^ (L3 and L4) lines. Arrowheads point to 46 kDa corresponding to the endogenous SIMK protein and to 72 kDa corresponding to the fusion GFP-SIMK protein (**A-B**). Relative SIMK (**C-D**) and GFP-SIMK (**D**) protein levels in roots and leaves of control, transgenic, and double transgenic plants are shown in graphs under respective PVDF membranes. Lowercase letters indicate statistical significance between lines according to one-way ANOVA with post-hoc Tukey HSD test (*P* < 0.05). Error bars show ± SD

Next, the SIMK and GFP-SIMK (Fig. 5A-B) proteins were detected in the newly prepared transgenic double lines SIMKKi^tagRFP−TUA6^ and GFP-SIMK^tagRFP−TUA6^. Endogenous SIMK protein with a molecular mass around 46 kDa and fusion GFP-SIMK protein with a molecular mass around 72 kDa were quantified (Fig. 5C-D). Relative SIMK abundance was strongly decreased in roots of the SIMKKi^tagRFP−TUA6^ line to approximately 5.83% in L1 and 5.72% in L2 lines and to 3.11% in L1 and 5.78% in L2 lines in leaves (Fig. 5C). The GFP-SIMK fusion protein was detected in the transgenic tagRFP-TUA6 carrying the *35S::GFP:SIMK* construct (Fig. 5B). Still, compared to the GFP-SIMK line, its abundance was decreased in both roots and leaves (Fig. 5D).

### Above-ground and root hairs phenotypes

**Fig. 6:**
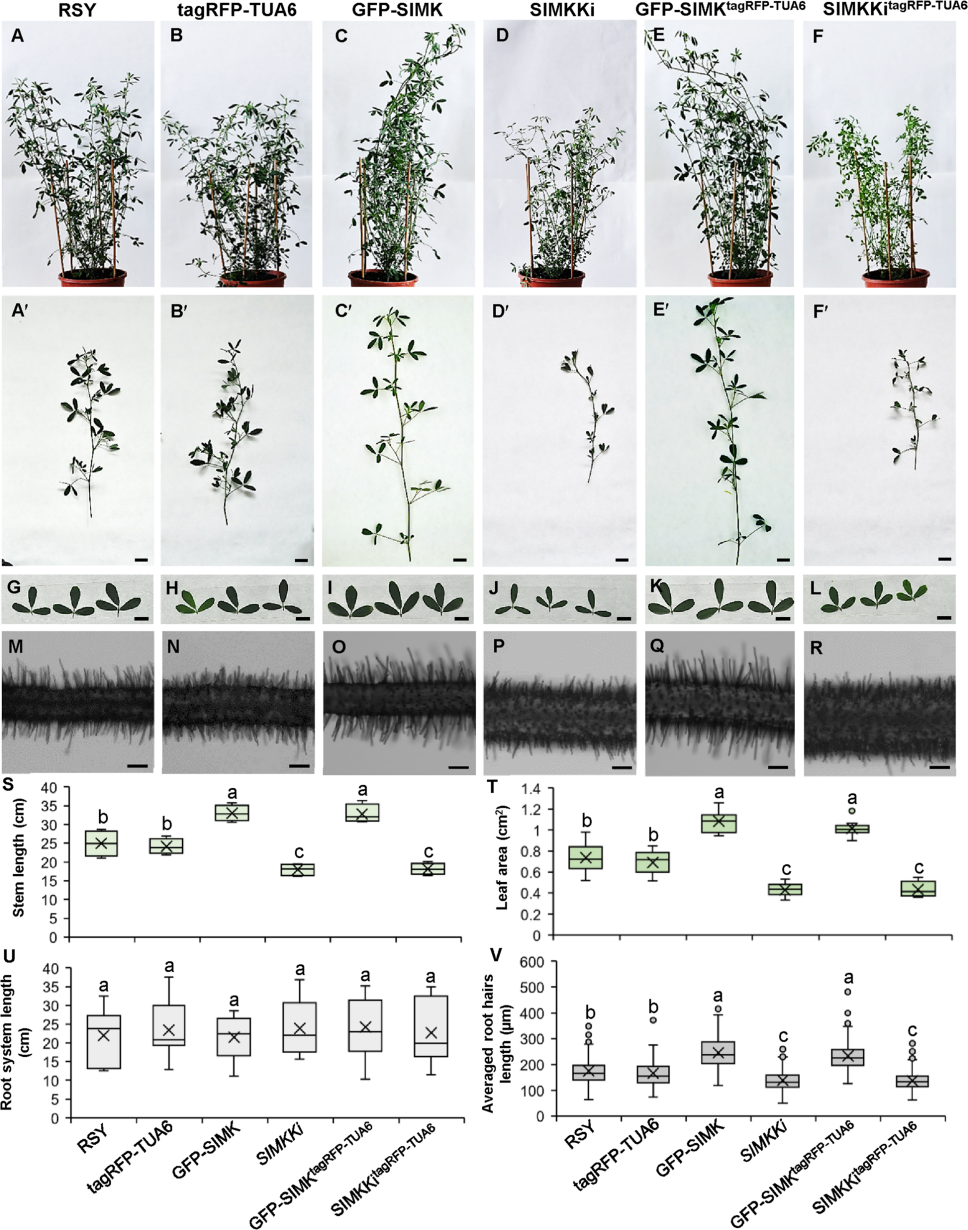
Above-ground part and root hair phenotypes in alfalfa transgenic lines co-expressing tagRFP-TUA6 microtubular molecular marker. Representative images of above-ground parts of mature plants regrown in pots in control RSY (**A**), transgenic tagRFP-TUA6 (**B**), GFP-SIMK (**C**), SIMKKi (**D**), and transgenic double GFP-SIMK^tagRFP−TUA6^ (**E**), and SIMKKi^tagRFP−TUA6^ (**F**) lines. Representative images of stems in control RSY (**A′**), transgenic tagRFP-TUA6 (**B′**), GFP-SIMK (**C′**), SIMKKi (**D′**), and transgenic double GFP-SIMK^tagRFP−TUA6^ (**E′**), and SIMKKi^tagRFP−TUA6^ (**F′**) lines. Representative images of leaves in control RSY (**G**), transgenic tagRFP-TUA6 (**H**), GFP-SIMK (**I**), SIMKKi (**J**), and transgenic double GFP-SIMK^tagRFP−TUA6^ (**K**), and SIMKKi^tagRFP−TUA6^ (**L**) lines. Representative images of root hairs in control RSY (**M**), transgenic tagRFP-TUA6 (**N**), GFP-SIMK (**O**), SIMKKi (**P**), and transgenic double GFP-SIMK^tagRFP−TUA6^ (**Q**), and SIMKKi^tagRFP−TUA6^ (**R**) lines. Box plot graphs depicting comparison in stem length (**S**; *N* = 10 for all analyzed lines), leaf area (**T;** *N* = 10 for all analyzed lines), root length (**U**; *N* = 6 for RSY; *N* = 9 for tagRFP-TUA6; *N* = 6 for GFP-SIMK, *N* = 7 for SIMKKi, *N* = 13 for GFP-SIMK^tagRFP−TUA6^; *N* = 8 for SIMKKi^tagRFP−TUA6^), and root hairs length (**V**; *N* = 501; *N* = 456; N = 463, *N* = 613, *N* = 488; *N* = 567) of indicated lines. Lowercase letters indicate statistical significance between lines according to one-way ANOVA with post-hoc Tukey HSD test (*P* < 0.05). Scale bar = 2 cm (A′-F′), 1 cm (G-L), and 200 µm (M-R)

Based on the differences in microtubule organization in different alfalfa organs and tissues, mainly stems and leaves, newly established transgenic double lines were subjected to phenotypic characterization. The above-ground part of alfalfa plants was examined in stably transformed double lines with genetically manipulated SIMK carrying the microtubular molecular marker, GFP-SIMK^tagRFP−TUA6^ and SIMKKi^tagRFP−TUA6^, together with the control RSY and transgenic tagRFP-TUA6, GFP-SIMK, and SIMKKi lines (Fig. 6A-L). Documentation of plants growing in pots 30 days after cutting revealed a smaller habitus of the above-ground part in the SIMKKi (Fig. 6D) and SIMKKi^tagRFP−TUA6^ (Fig. 6F) lines in comparison with control RSY and tagRFP-TUA6 lines (Fig. 6A-B). On the other hand, the above-ground part of the GFP-SIMK (Fig. 6C) and GFP-SIMK^tagRFP−TUA6^ (Fig. 6E) lines formed a more robust and bushy habitus. In quantitative terms, the GFP-SIMK (Fig. 6C′) and GFP-SIMK^tagRFP−TUA6^ (Fig. 6E′) lines produced significantly longer shoots in comparison with control RSY (Fig. 6A′) and transgenic tagRFP-TUA6 (Fig. 6B′) plants. On the contrary, the SIMKKi (Fig. 6D′) and the transgenic double SIMKKi^tagRFP−TUA6^ (Fig. 6F′) lines developed significantly smaller shoots (Fig. 6S). Considering the leaf size, the SIMKKi (Fig. 6J) and transgenic double SIMKKi^tagRFP−TUA6^ (Fig. 6L) lines formed significantly smaller leaves in comparison with control RSY (Fig. 6G) and tagRFP-TUA6 (Fig. 6H) lines, while the GFP-SIMK (Fig. 6I) and GFP-SIMK^tagRFP−TUA6^ (Fig. 6K) lines developed significantly larger leaves (Fig. 6T). Microtubules are crucial for cell expansion and morphogenesis and might influence leaf size and shape. Deviations in their organization in the transgenic SIMKKi^tagRFP−TUA6^ line (Fig. 2) could lead to abnormal leaf development. Besides that, root length and root hair phenotypes were examined. Regarding the length of the whole root system, no significant differences were observed among the analyzed lines (Fig. 6U). The length of root hairs with terminated tip growth within the root differentiation zone was measured. In control RSY plants, the root hair length median value was 174 µm, and in the transgenic tagRFP-TUA6 line, the median value was similar, about 165 µm (Fig. 6M-N; V). In contrast, the transgenic double line GFP-SIMK^tagRFP−TUA6^ showed an increase in root hair length median to 232 µm (Fig. 6Q; V), similarly to the GFP-SIMK line with a median value of 245 µm (Fig. 6O; V), while the SIMKKi^tagRFP−TUA6^ line showed a decrease in median value to 137 µm (Fig. 6R; V), similarly to the SIMKKi line with a median value of 139 µm (Fig. 6P; V).

## Discussion

It has been demonstrated that adaptation to changing environments via MAPK cascades regulates cytoskeletal rearrangements, which is primarily achieved through MAPK-mediated phosphorylation of target cytoskeleton-associated proteins (Komis et al. 2018; Vavrdová et al. 2019b). Moreover, cells utilize the cytoskeleton as a sensor of developmental and environmental signals (Nick 2013; Chen et al. 2016; Hamant et al. 2019). It has become obvious that the crosstalk between the plant cytoskeleton and MAPK signaling pathways is necessary to control crucial cellular activities. To study the impact of SIMK abundance on microtubules in the legume crop species, alfalfa, we prepared transgenic double lines with genetically manipulated SIMK co-expressing the microtubular molecular marker tagRFP-TUA6, which enabled the study of SIMK-dependent microtubule organization in different plant organs and tissues depending on SIMK abundance.

In the SIMKKi^tagRFP−TUA6^ line, microtubule atypical branching and disorganization were prominent, especially in leaf epidermal cells and secondary leaf veins, which also tended to form right-handed helical cell files. Additionally, we observed the aberrant positioning of phragmoplasts and shifted CDPs, resulting in abnormal patterns and variable sizes of cell files in the root tip of the SIMKKi^tagRFP−TUA6^ line. These phenotypes exhibited a resemblance to those previously described in the Arabidopsis *mpk6* mutant for SIMK orthologue MPK6 (Müller et al. 2010) and Arabidopsis *ΔNyda1* mutant of the YODA pathway employing MPK6 (Smékalová et al. 2014). MPK6 depletion in Arabidopsis *mpk6* mutants caused microtubule atypical bundling and aberrant cell file formation in the root as a result of cell division plane misorientations (Müller et al. 2010). *ΔNyda1* showed ectopic cell divisions in the central cylinder and pericycle, but also vigorous twisting of all root tissues (Smékalová et al. 2014). The pathway downstream of YODA has also been shown to affect cortical microtubule organization (Smékalová et al. 2014). MAPKs have already been shown to associate with microtubules in plants. Previous studies have demonstrated that MPK6 has a dominant nuclear localization (Smékalová et al. 2014), but a fraction may associate with the plasma membrane and cytoplasmic vesicular structures (Müller et al. 2010). In addition, specific associations of Arabidopsis MPK6 with cortical microtubules (Vavrdová et al. 2019a), pre-prophase bands, phragmoplasts, and cell plates (Müller et al. 2010; Smékalová et al. 2014; Kohoutová et al. 2015), as well as its colocalization with these structures, were shown. Here, subcellular localization of GFP-SIMK in phragmoplasts and its association with cortical microtubules was observed in living cells and immunolabeling revealed an association of alfalfa SIMK with phragmoplasts and cortical microtubules in roots of control RSY and GFP-SIMK lines, which was not the case for low-abundant SIMK in the transgenic SIMKKi line. Previously, considerably decreased activity of SIMK was detected in the SIMKKi line, while in the overexpression GFP-SIMK line, SIMK activity was strongly increased (Hrbáčková et al. 2021).

Quantification of the tubulin cytoskeleton between the compared double lines revealed differences in their cortical microtubule organization, mainly in leaves and stems. The skewness of the fluorescence intensity distribution is considered to be an indicator of filament bundling in cells (Higaki et al. 2010). Both tagRFP-TUA6 and overexpression GFP-SIMK^tagRFP−TUA6^ lines exhibited a significantly higher degree of bundling compared to the SIMKKi^tagRFP−TUA6^ line with downregulated *SIMK*, primarily in the stem, leaf epidermis, and leaf secondary veins. Microtubule bundles are suggested to have greater angular and positional stability than single microtubules, also affect microtubule dynamics in plant cells (Ehrhardt and Shaw 2006; Bratman and Chang 2008), and have important organizational and functional properties. Among microtubule bundlers shown to be phosphorylated by MAPKs are MAP65-1, MAP65-2, and MAP65-3 (Beck et al. 2010; Kosetsu et al. 2010; Sasabe et al. 2011; Smékalová et al. 2014). Arabidopsis MAP65-1 is phosphorylated by both MPK4 and MPK6 in vitro (Smertenko et al. 2006), although it is a preferred substrate for MPK4 (Zhang et al. 2016; Jiang et al. 2022). The physical interaction between MAP65-1 and MPK6 is relatively weak as revealed by co-immunoprecipitation assays (Smékalová et al. 2014). In addition, MAP65-2 and MAP65-3 have also been shown to be phosphorylated by MPK4 (Kosetsu et al. 2010; Sasabe et al. 2011). Since the extent of microtubule bundling is higher in the tagRFP-TUA6 and GFP-SIMK^tagRFP−TUA6^ lines and SIMK associates with cortical microtubules in addition to their branching points also at microtubule bundles, MAP65 might be potentially also targeted by SIMK. Importantly, observed distribution of GFP-SIMK associated with bundled microtubules (Suppl. Fig. S4D-L) resembled a distribution pattern of MAP65-2 dimers along bundled microtubules acquired by advanced single-molecule photo-activation localization microscopy (PALM) method (Vavrdová et al. 2019b). MPK6 also interacts with γ-tubulin (Kohoutová et al. 2015), which plays an important role in microtubule nucleation, organization, and branching by acting as a nucleating factor (Murata et al. 2005; Binarová et al. 2006; Pastuglia et al. 2006). Possible association of alfalfa SIMK with γ-tubulin could be anticipated from the localization of SIMK at microtubule branching points. However, despite the close association of γ-tubulin with MPK6 and active phosphorylated ERK1 shown in Arabidopsis, phosphorylation of γ-tubulin or GAMMA-TUBULIN COMPLEX COMPONENT 4 protein by active MPK6 was not detected (Kohoutová et al. 2015). It was demonstrated that mutations in the γ-tubulin-containing complex can lead to helical growth and abnormal microtubule branching (Nakamura and Hashimoto 2009). Here, we observed similar phenotypes in the SIMKKi^tagRFP−TUA6^ line, showing microtubule disorganization and atypical branching in leaf epidermis and secondary vein cells. EB1c is a protein that accumulates at microtubule plus-ends and regulates their dynamics and polarization. It is a direct target of MPK6, regulated by phosphorylation during plant cytokinesis (Kohoutová et al. 2015). It was demonstrated that MPK6 phosphorylates EB1c and has a role in maintaining regular planes of cell division under stress conditions (Kohoutová et al. 2015). EB1c localizes to the nucleus in non-dividing root meristematic cells, while it decorates mitotic microtubular structures during cell division (Novák et al. 2016; Komis et al. 2018). Moreover, EB1c contributes to the organization of cortical microtubules in growing plant epidermal cells (Molines et al. 2018). In EB1-deficient Arabidopsis plants, reduced microtubule bundling, partial microtubule disorganization, and lower orderliness were observed (Molines et al. 2018). Besides impaired bundling and shifted CDPs, the anisotropy of cortical microtubules in the transgenic SIMKKi^tagRFP−TUA6^ was significantly lower in most of the analyzed cell types when compared to the GFP-SIMK^tagRFP−TUA6^ and tagRFP-TUA6 lines, indicating disordered microtubules upon *SIMK* downregulation. It should be noted that an anisotropy value of 1 can never be achieved with biological samples, and our results showed the highest anisotropy of 0.3–0.4, which is consistent with published values for microtubules (Boudaoud et al. 2014). Microtubule organization is likely to be mediated by MAPs such as EB1c and MAP65, and SIMK might phosphorylate and regulate some of them. Therefore, SIMK-mediated phosphorylation of target cytoskeleton-associated proteins influencing the microtubule organization in alfalfa might be directly dependent on SIMK abundance.

Moreover, purely random orientation of cortical microtubules was evident in the SIMKKi^tagRFP−TUA6^ line in secondary leaf veins and leaf epidermal cells that were visibly smaller with reduced lobe outgrowths. Studies on the cytoskeletal basis of leaf epidermal pavement cell morphogenesis have shown that cortical microtubules mediate cellulose deposition along the adaxial–abaxial axis in the internal cell wall (Zhao et al. 2020; Schneider et al. 2022), which tend cortical microtubules to be organized into parallel bundles in the indentations of young leaf pavement cells. This arrangement is required for lobe formation and is regulated, among others, by various MAPs (Chen et al. 2022). Parallel bundles of microtubules were observed in leaf epidermal cells of the GFP-SIMK^tagRFP−TUA6^ and tagRFP-TUA6 lines, while in the SIMKKi^tagRFP−TUA6^ line, microtubules were disorganized. Lobe formation depends on coordinated activities of microtubules and actin filaments, both of which are controlled by ROPs (Fu et al. 2005). A simplified model of the ROP-dependent polarity pathway in tip-growing cells, proposed by Ou and Yi (2022), shows four different types of ROP effectors, one of which targets cytoplasmic regulators (e.g. kinases) to regulate RopGEFs and ROP activity. Moreover, microtubules control the oriented deposition of cellulose microfibrils in the cell wall, which is essential for the direction of cell growth. Microtubule random orientation could lead to imprecise control of cellulose deposition, which could significantly affect plant morphogenesis, including the development of plant tissues and organs. Phenotypic analysis revealed differences in the above-ground part and root system between the two lines, SIMKKi^tagRFP−TUA6^ and GFP-SIMK^tagRFP−TUA6^. These differences may be linked to altered microtubule organization in the SIMKKi^tagRFP−TUA6^ line, demonstrated by microtubule branching, disorganization, and reduced anisotropy and bundling mainly in the above-ground plant part. The SIMKKi^tagRFP−TUA6^ line formed significantly shorter shoots, smaller leaves, and shorter root hairs, whereas the introduction of the *35S::GFP:SIMK* construct into the tagRFP-TUA6 line promoted opposite phenotypes. This is confirmed by a previous study using only the transgenic GFP-SIMK and SIMKKi lines, where Hrbáčková et al. (2021) showed that overexpression of alfalfa SIMK promotes root hair growth and shoot biomass formation. Plant cell morphogenesis is affected by both the microtubule and actin cytoskeletal networks and the signaling mechanisms that control their organization. As organ shape is controlled by both directed cell expansion and cell division, the regulation of microtubule organization is essential in these processes to determine cell morphology. Importantly, this might be orchestrated by MAPKs. This is likely implicated in other aspects of plant ontogenesis, where cell morphology relies on precise regulation and control of microtubule arrangements. Among others, good example is plant reaction to microtubule inhibitors that are utilized as important tools in weed management and crop protection. These compounds are widely used in agriculture. Microtubule-inhibiting herbicides like dinitroanilines (trifluralin, pendimethalin) and phosphoric amides (amiprophos-methyl), bind to tubulin heterodimers. Created herbicide-tubulin complexes inhibit polymerization of microtubules (Chen et al. 2021), thus preventing microtubule arrangements to their typical arrays. Consequently, sensitive weed species are eliminated by disrupting the formation and function of microtubules in all affected proliferating cells. However, this broadly applied approach is currently compromised by selection of herbicide-resistant weeds (Boyd et al. 2022; Torra and Alcántara-de la Cruz 2022; Bobadilla and Tranel 2024). It requires a development of new strategies for weed management, including a design of new microtubule-inhibiting herbicides. To their thorough testing on the level of plant cell morphogenesis and development in vivo, based on the microtubule cytoskeletal network as a target and including the MAPK-based signaling mechanisms that control their organization, new alfalfa lines with microtubule molecular markers must be available.

Since the regulation of cytoskeleton rearrangements by MAPK signaling cascades can influence plant development, it could be suggested that SIMK can directly regulate some MAPs and thus indirectly affect developmental processes in alfalfa, depending on microtubules. In conclusion, these results indicate that SIMK genetic manipulation has an important effect on microtubule organization in alfalfa, but further studies will be necessary to unveil the interplay between SIMK-dependent signaling to microtubules, most likely through some MAPs, in a functional context. Therefore, our double transgenic alfalfa lines represent a reliable tool for future progress in the establishment of precision agriculture, including targeted weed control by using microtubule inhibitors.

## Materials and methods

### Plant material

To prepare alfalfa stable transgenic double lines with genetically manipulated SIMK co-expressing cytoskeletal marker for microtubules, alfalfa tagRFP-TUA6 and *SIMKK-RNAi* (SIMKKi; Hrbáčková et al. 2021) lines cultivated in vivo in the environmental chamber at constant conditions (21 °C, 70% humidity, 16 h-light/8 h-dark cycle) were used for *Agrobacterium tumefaciens-*mediated leaf explant stable transformation.

### Alfalfa stable transformation

Leaves of alfalfa transgenic tagRFP-TUA6 and SIMKKi lines were surface sterilized and transformed with *A. tumefaciens* strain GV3101 carrying either *35S::GFP:SIMK* (N-terminal fusion of enhanced green fluorescent protein to SIMK; Hrbáčková et al. 2021) or *35S::tagRFP:TUA6* (N-terminal fusion of monomeric red fluorescent protein to TUBULIN ALPHA 6; Murata et al. 2013) construct according to the previously published protocol (Samac and Austin-Phillips, 2006). Leaves of alfalfa plants were cut in half, wounded on the surface with a sterile scalpel blade, and incubated for 30 min in the dark with the overnight *Agrobacterium* culture reaching OD_600_ = 0.7. Leaf explants were briefly blotted on sterile filter paper to remove excess bacterial solution and calli, somatic embryos, and regenerated stably transformed plantlets were induced by cultivation on appropriate media in the environmental chamber under constant conditions. Phosphinothricin (15 μg/ml; for *35S::GFP:SIMK*) or kanamycin (25 µg/ml; for *35S::tagRFP:TUA6*) were used as a selection markers *in planta*, together with ticarcillin (500 μg/ml) to inhibit *Agrobacterium* growth after transformation. Regenerated plants of transgenic double lines were maintained on MS media and selected for the presence of either GFP-SIMK or tagRFP-TUA6 fusion proteins by confocal laser scanning microscope LSM 710 (Carl Zeiss, Germany). Alfalfa transgenic double lines were further propagated via somatic embryogenesis, and from each double line, two independent lines were selected, termed SIMKKi^tagRFP−TUA6^ L1 and L2, and GFP-SIMK^tagRFP−TUA6^ L3 and L4.

### Immunolocalization of microtubules and SIMK in root whole mounts

Immunolocalization of microtubules and SIMK in root whole mounts was performed as previously described (Tichá et al. 2020; Hlaváčková et al. 2023). Root tips used for chemical fixation were derived from *M. sativa* wild-type RSY (control) and transgenic SIMKKi, and GFP-SIMK lines. Samples were first immunolabeled with rabbit anti-MPK6 (Sigma, Life Science, USA) primary antibody diluted 1:750 in 2.5% (w/v) BSA. To enhance antibody penetration, samples were subjected to vacuum infiltration (3 × 5 min), followed by overnight incubation at 4 °C. After washing with PBS were samples incubated with appropriate secondary antibodies: control RSY and SIMKKi samples with Alexa Fluor 555-conjugated goat anti-rabbit (Abcam, UK) and GFP-SIMK samples with Alexa Fluor 488-conjugated goat anti-rabbit secondary antibody (Invitrogen, USA), both diluted 1:500 in 2.5% (w/v) BSA in PBS, for 2 h at 37 °C. Following extensive washing with PBS and blocking in 5% (w/v) BSA in PBS, samples were subsequently immunolabeled with rat anti-α-TUBULIN (clone YOL1/34; ABD Serotec) primary antibody diluted 1:350 in 2.5% (w/v) BSA in PBS, with overnight incubation at 4 °C. Following extensive washing in PBS and blocking in 5% (w/v) BSA, samples were incubated with appropriate secondary antibodies: control RSY and SIMKKi with Alexa Fluor 488-conjugated goat anti-rat secondary antibody (Invitrogen, USA) and GFP-SIMK samples with Alexa Fluor 647-conjugated goat anti-rat secondary antibody (Abcam, UK), both diluted 1:500 in 2.5% (w/v) BSA in PBS, for 2 h at 37 °C. DAPI was used for nuclear counterstaining. Image acquisition was conducted using either a Zeiss LSM 710 confocal laser scanning microscope (Carl Zeiss, Germany) or a Zeiss LSM 880 Airyscan system equipped with a 32-channel GaAsP detector (Carl Zeiss, Germany). Excitation was performed using laser lines at 405 nm (DAPI), 488 nm (Alexa Fluor 488), 561 nm (Alexa Fluor 555), and 631 nm (Alexa Fluor 647), generated by argon, HeNe, diode, and diode-pumped solid-state lasers, respectively. Image processing was performed using ZEN 2014 software (Carl Zeiss, Germany), Adobe Photoshop 6.0/CS, and Microsoft PowerPoint.

### Immunoblot analysis

Immunoblot analysis was performed as described in Takáč et al. (2017). For this analysis, 18-day-old plants of double transgenic GFP-SIMK^tagRFP−TUA6^ L3-L4 and SIMKKi^tagRFP−TUA6^ L1-L2 lines, control RSY and transgenic SIMKKi, GFP-SIMK, and tagRFP-TUA6 lines were used. Roots and leaves were homogenized using liquid nitrogen into a fine powder. Proteins were extracted in E-buffer [50 mM HEPES (pH 7.5), 75 mM NaCl, 1 mM EGTA, 1 mM MgCl_2_, 1 mM NaF, 10% (v/v) glycerol, Complete™ EDTA-free protease inhibitor, and PhosSTOP™ phosphatase inhibitor cocktails (both from Roche, Basel, Switzerland)] and centrifuged (13000 g, 20 min, 4 °C). Concentration of proteins was measured by Bradford assay (Bio-Rad), supernatants were mixed with 4 × concentrated Laemmli buffer [final concentration 62.5 mM Tris–HCl (pH 6.8), 2% (w/v) SDS, 10% (v/v) glycerol, 300 mM 2-mercaptoethanol], and boiled for 5 min at 95 °C. An equal amount of proteins (30 µg) was separated on 12% TGX Stain-FreeTM Fast-Cast™ gels (Bio-Rad). Separated proteins were transferred to polyvinylidene difluoride (PVDF) membrane (GE Healthcare) using a wet tank unit (Bio-Rad) with Tris/glycine/methanol transfer buffer at 24 V and 4 °C overnight. Nonspecific epitopes were blocked by incubating membranes in 4% (w/v) BSA and 4% (w/v) low-fat dry milk in Tris-buffered-saline with Tween 20 (TBS-T, 100 mM Tris–HCl; 150 mM NaCl; 0.1% (v/v) Tween 20; pH 7.4) when using anti-AtMPK6 and anti-RFP antibodies, or in 4% (w/v) low-fat dry milk when using anti-TUBULIN α antibody at 4 °C overnight. Subsequently, membranes were incubated with anti-AtMPK6 (1:15000, Sigma, Life Science, USA), anti-RFP (1:2000, Evrogen), and anti-TUBULIN α (1:1000, Agrisera) primary antibodies in TBST-T containing 1% (w/v) BSA at 4 °C overnight. After repeated washing with TBST-T, membranes were incubated with horseradish peroxidase (HRP)-conjugated anti-rabbit or anti-mouse IgG secondary antibody diluted in TBS-T (1:5000) containing 1% (w/v) BSA for 2 h. The signal was developed after three washing steps with TBS-T by using the Clarity Western ECL (Enhanced chemiluminescence) substrate (Bio-Rad) and detected on the Chemidoc MP documentation system (Bio-Rad). Band optical densities were measured using Image Lab software (Bio-Rad). All band densities were normalised according to loading controls.

### In vivo imaging

For the microscopic analysis of microtubule organization, alfalfa tagRFP-TUA6, GFP-SIMK^tagRFP−TUA6^, and SIMKKi^tagRFP−TUA6^ plants were regenerated by somatic embryogenesis. Small plantlets were placed on a slide with a chamber made from double-sided tape into a drop of liquid MS medium, covered with a coverslip, and sealed with parafilm. Images were acquired by the Zeiss LSM 710 platform (Carl Zeiss, Germany) equipped with Plan-Apochromat 40 × 1.4 Oil DIC M27 objective. Samples were imaged with excitation laser lines at 488 nm for GFP (to detect GFP-SIMK fusion protein), 561 nm for mRFP (to detect tagRFP-TUA6) fusion protein, and appropriate emission spectra.

To delineate the root epidermal cell periphery of control RSY and transgenic SIMKKi and GFP-SIMK lines, the vital red-fluorescent styryl dye FM4-64 was used. Isolated root tips were stained by incubation in 4 µM FM4-64 prepared in liquid MS medium for 45–60 min. At the beginning of the incubation, a weak vacuum was used (3 × 5 min). Image acquisition was conducted using Zeiss LSM 710 confocal laser scanning microscope (Carl Zeiss, Germany) equipped with Plan-Apochromat 20 × 0.8 M27 objective. Excitation was performed using laser lines at 488 nm and emission spectra 493–560 nm (GFP-SIMK) and 610–750 nm (FM4-64).

### Image acquisition and processing

The image acquisition, post-processing, quantitative analysis, maximum intensity projections from individual z-stacks, and subset creation of all fluorescence images were performed using Zeiss ZEN software (Black and Blue versions, Carl Zeiss, Germany). Although quantification of fluorescence intensities is not influenced by post-acquisition look-up table (LUT) intensity adjustments, all images used for quantitative analyses of tubulin cytoskeleton were acquired under the same imaging conditions, with brightness and contrast adjusted uniformly for all fluorescence images. The same laser attenuation values for all laser lines were set before the acquisition, and the thickness of individual optical sections was optimized according to Nyquist criteria. The pinhole sizes for green (GFP) and red (mRFP) channels were matched, and the range of detection was appropriately adjusted to ensure the separation of emission wavelengths and to prevent fluorescence spectral bleed-through. If necessary, denoising of microscopy images was performed by using the Arivis Vision4D 3.0.1 software (Arivis AG, Rostock, Germany). Based on the lower abundance and fluorescence intensity of the tagRFP-TUA6 fusion protein in the transgenic double SIMKKi^tagRFP−TUA6^ line, the brightness and contrast were across lines unevenly adjusted only for the preparation of representative images (Fig. 2) to ensure sufficient visualization of microtubules without oversaturation or underexposure. Images exported from ZEN software were then assembled into final figure plates using Microsoft PowerPoint.

### Semi-quantitative analysis of the fluorescence intensity distribution

Selected data obtained from the confocal live-cell imaging of the transgenic double GFP-SIMK^tagRFP−TUA6^ line from cells of primary and secondary leaf veins were semi-quantitatively evaluated by profile measurements to study the association of GFP-SIMK with cortical microtubules. Distribution of GFP-SIMK and tagRFP-TUA6 fluorescence intensity was determined by profile measurement. Intensity profiles were quantified across cortical microtubules in the direction indicated in appropriate images by white dashed arrows. This analysis was done using a measure function of Zeiss ZEN 2011 software (Black version) from single confocal optical sections.

### Quantification of the tubulin cytoskeleton

Microtubule organization was quantitatively addressed by parameters of average angle orientation, anisotropy, and skewness. Ordering of cortical microtubules was measured as the average angle of their orientation through the FibrilTool macro as described previously (Boudaoud et al. 2014). The degree of cortical microtubule ordering was quantitatively assessed by anisotropy, which is defined as the existence of a dominant microtubule orientation as compared to the main cell axis (Landrein and Hamant 2013). Microtubule anisotropy was demonstrated with the FibrilTool macro, and regarding the anisotropy score, the following convention is used: 0 for no order (purely isotropic arrays) and 1 for perfectly ordered (purely anisotropic arrays). Skewness was measured to assess the extent of microtubule bundling. This parameter practically evaluates how much the fluorescence intensity of each given pixel in the image deviates from the mean value, or, in other words, how uniformly the sample is labeled. When the sample is uniformly labeled (i.e., when microtubules are uniformly distributed in the cortical cytoplasm), the skewness value is 0. When fluorescence distribution is non-uniform (i.e., when there are distinct dark and fluorescent spaces), and depending on how much non-uniform it is (i.e., dark and fluorescent structures are unevenly distributed), then it is considered to be skewed and has values different than 0. The FibrilTool macro was applied to ROIs drawn using the Polygon tool of ImageJ, delineating the circumference of fully visible cells. Cell borders with frequently saturated edges, which could be falsely added to the result, were avoided. Orientation of CDPs was measured in Zeiss ZEN software (Blue edition). Quantification of CDPs orientation was performed from roots of alfalfa RSY, tagRFP-TUA6, SIMKKi, SIMKKi^tagRFP−TUA6^, GFP-SIMK, and GFP-SIMK^tagRFP−TUA6^ lines. The angular positioning of cross-walls with respect to the root axis was measured on microscopic images of root cells and angles were measured in root epidermal cell files. Obtained data were divided into two categories according to the measured angles: correctly oriented CDPs (90° ± 5%) and incorrectly oriented CDPs (less than 85° and more than 95°). Graphs depicting angle distributions and relative frequencies of angle distributions were prepared in GraphPad Prism 10.5.0 and Microsoft Excel softwares, respectively. For tagRFP-TUA6, SIMKKi^tagRFP−TUA6^, and GFP-SIMK^tagRFP−TUA6^ lines, rose plots were generated in Python 3.10 using angles and displayed in polar histograms to illustrate orientation patterns within each line.

### Phenotypic analysis

Plants of alfalfa wild-type RSY, transgenic tagRFP-TUA6, and double transgenic SIMKKi^tagRFP−TUA6^ and GFP-SIMK^tagRFP−TUA6^ lines were used for phenotypic analysis of root hair length. Through somatic embryogenesis regenerated 18-day-old plants of control and transgenic lines growing on MS medium in Petri dishes were used for root hair imaging with Axio Zoom.V16 stereomicroscope (Carl Zeiss, Germany). Root hairs and root system length were measured in the ImageJ software. Pictures of the whole above-ground parts, individual shoots, and trifoliate leaves from wild-type RSY, transgenic tagRFP-TUA6, and double transgenic SIMKKi^tagRFP−TUA6^ and GFP-SIMK^tagRFP−TUA6^ plants regrown in pots 30 days after shoot cutting (Gou et al. 2018) were acquired by digital camera (Nikon D5000, Japan). Individual shoots were then detached from the plants, and stem length (in cm) and total leaf area (left, right, and apical leaflet together) on the 3rd leaf of one stem were measured in ImageJ software and quantified.

### Statistical analysis

Statistical parameters of performed experiments, number of samples (n), and type of statistical test are included in the figure legends. Graphs were prepared in Microsoft Excel, Python 3.10, or GraphPad Prism 10.5.0 and finalized using PowerPoint software. Statistical significance (*P* < 0.05) was determined using the Statistica 13.4.0 software (TIBCO Software Inc., Palo Alto, CA, USA) by one-way ANOVA with post-hoc Tukey HSD test (*P* < 0.05). Statistically significant differences (*P* < 0.05) in angle dispersion between alfalfa lines were determined by *F*-test. When box plots are used, they display the first and third quartiles split by the median, the crosses indicate the mean values, and whiskers extend to include the max/min values.

## Supplementary Information

Supplementary Material 1: Fig. S1 Co-immunolocalization of phragmoplast microtubules and SIMK in alfalfa control and transgenic lines. Fig. S2 Cell division planes (CDPs) orientation in root meristems of alfalfa control and transgenic lines. Fig. S3 Association of SIMK with branching points at cortical microtubules in alfalfa control and transgenic lines. Fig. S4 SIMK association with bundling and branching points at cortical microtubules in alfalfa GFP-SIMK transgenic line. Table S1 Results of F-test determining significant differences in angle dispersion between two groups.

## Data Availability

The datasets used and/or analysed during the current study are available from the corresponding authors on reasonable request.

## Abbreviations

ANP2: Arabidopsis nucleus- and phragmoplast-localized kinase 2
ANP3: Arabidopsis nucleus- and phragmoplast-localized kinase 3
CDPs: Cell division planes
DIC: Differential interference contrast
EB1: End binding protein
EDTA: Ethylenediaminetetraacetic acid
EGTA: Ethylene glycol-bis(beta-aminoethyl ether)-N,N,N′,N′-tetraacetic acid
GFP: Green fluorescent protein
IgG: Imunoglobulin G
MAP65: Microtubule-associated protein 65-kDa
MAPKs: Mitogen-activated protein kinases
MAPs: Microtubule-associated proteins
MDP25: Microtubule-destabilizing protein 25
MMK2: Medicago MAPK2
NACK: Kinesin-like protein (Nicotiana)
NPK1: Nucleus- and phragmoplast-localized protein kinase 1
NQK1: Nicotiana kinase Q
NRK1: Nicotiana related kinase 1
PALM: Photo-activation localization microscopy
PVDF: Polyvinylidene difluoride
ROI: Region of interest
ROPs: Rho of plants
RSY: RegenSY (or Regenerated Synthetic), alfalfa genotype
SDS: Sodium dodecyl sulfate
SIMK: Stress-induced MAPK
SIMKK: Stress-induced MAPK kinase
SIMKKi: SIMKK-RNAi
tagRFP-TUA6: tag Red fluorescent protein-Tubulin alpha 6
YFP: Yellow fluorescent protein
