# Supplementary material for: Stress-induced MAPK (SIMK)-dependent organization of microtubules in alfalfa
Source: Mol Hortic. 2026 Jun 9;6:43. doi: 10.1186/s43897-026-00231-0 (PMC13248438; doi:10.1186/s43897-026-00231-0)
Supplement: Supplementary file 1 — Supplementary Material 1: Fig. S1 Co-immunolocalization of phragmoplast microtubules and SIMK in alfalfa control and transgenic lines. Fig. S2 Cell division planes (CDPs) orientation in root meristems of alfalfa control and transgenic lines. Fig. S3 Association of SIMK with branching points at cortical microtubules in alfalfa control and transgenic lines. Fig. S4 SIMK association with bundling and branching points at cortical microtubules in alfalfa GFP-SIMK transgenic line. Table S1 Results of F-test determining significant differences in angle dispersion between two groups. [file 43897_2026_231_MOESM1_ESM.pdf]

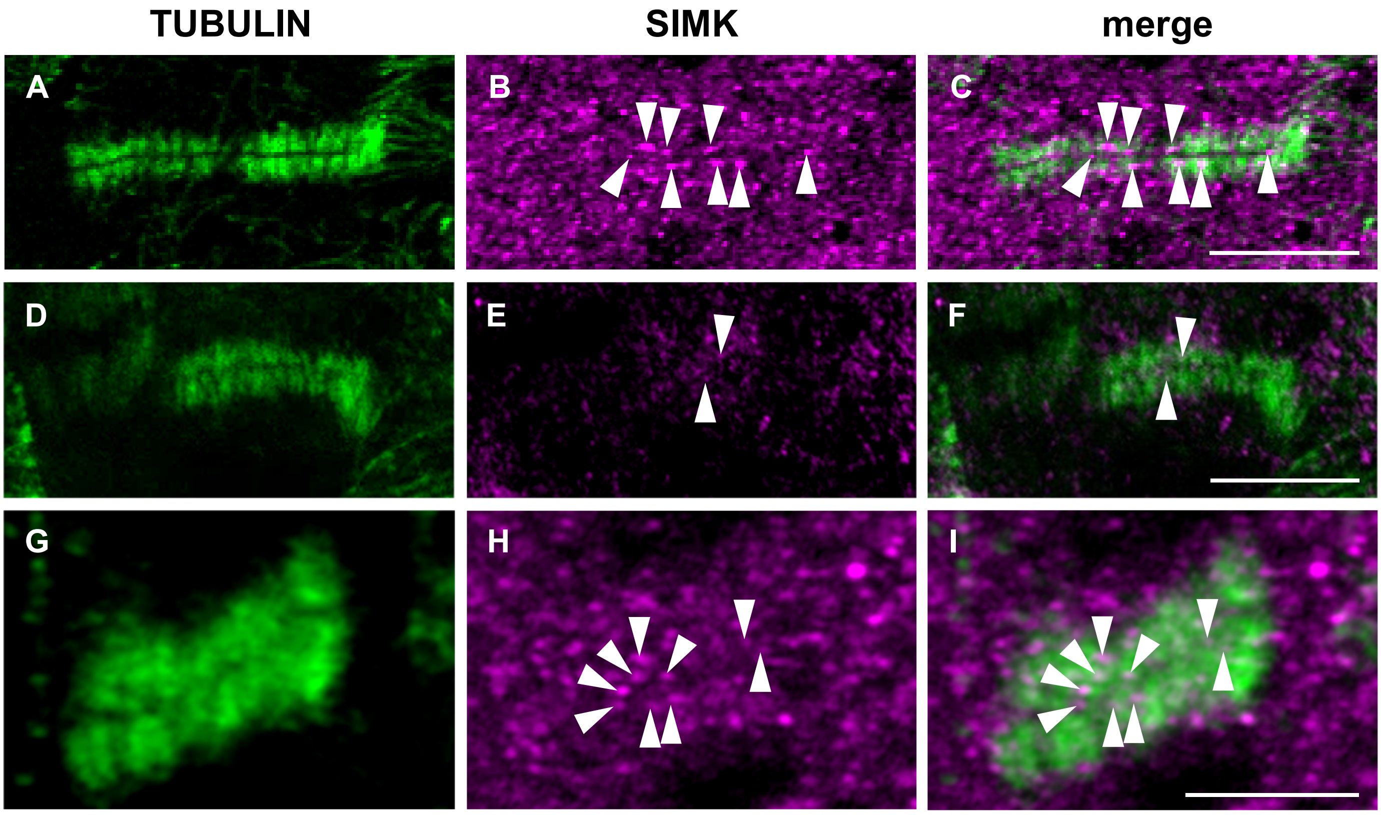


**Supplementary Fig. S1 Co-immunolocalization of phragmoplast microtubules and SIMK in alfalfa control and transgenic lines.** Representative images showing strong association of SIMK (magenta) with phragmoplast microtubules (green) in control RSY (**A-C**) and transgenic GFP-SIMK (**G-I**) lines. Arrowheads point to SIMK associated with middle part of the phragmoplasts, where phragmoplast microtubules by their + ends are oriented. Representative images showing very weak association of strongly downregulated SIMK with phragmoplast microtubules in the transgenic SIMKKi line (**D-F**). SIMK in the GFP-SIMK line (**G-I**) is pseudocolored in magenta and microtubules in green for consistency with the other images. Scale bar = 10 µm (A-F) and 5 µm (G-I).

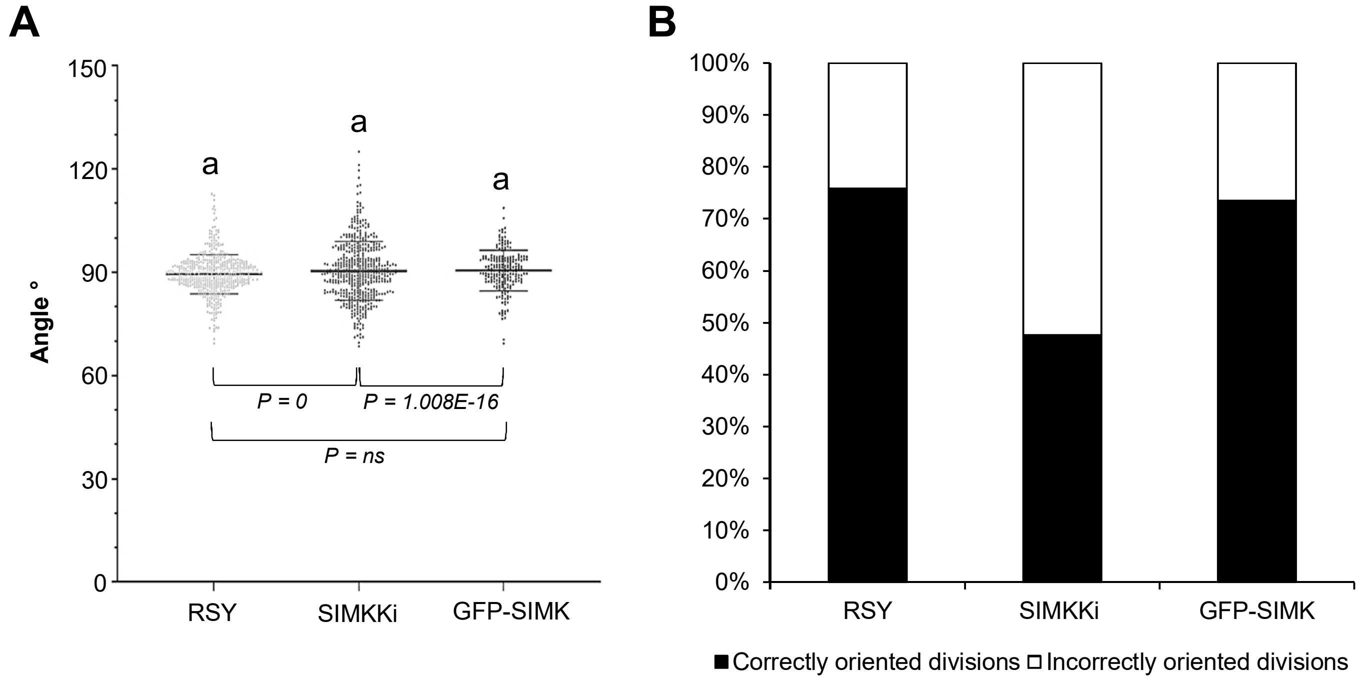


**Supplementary Fig. S2 Cell division planes (CDPs) orientation in root meristems of alfalfa control and transgenic lines.** Quantification of cross-wall orientation between neighbouring cells, indicating the division angle in epidermal cell files **(A)** of RSY (N=529), SIMKKi (N=492) and GFP-SIMK (N=626) lines. Scatter plots represent the distribution of individual division angle values and plot mean with SD. *P*-values in (**A**) show statistical significance (P<0.05) in angle dispersion between lines according to the *F*-test. Relative frequencies of angle distributions **(B)** in epidermal cell files of RSY (N=529), SIMKKi (N=492), and GFP-SIMK (N=626) lines, showing the percentage of correctly (90° ± 5°) and incorrectly (less than 85° and more than 95°) oriented CDPs. The epidermal cell periphery of isolated root tips was delineated using the vital red-fluorescent styryl dye FM4-64. Lowercase letters indicate statistical significance in mean angle between lines according to one-way ANOVA with post-hoc Tukey HSD test (P<0.05).

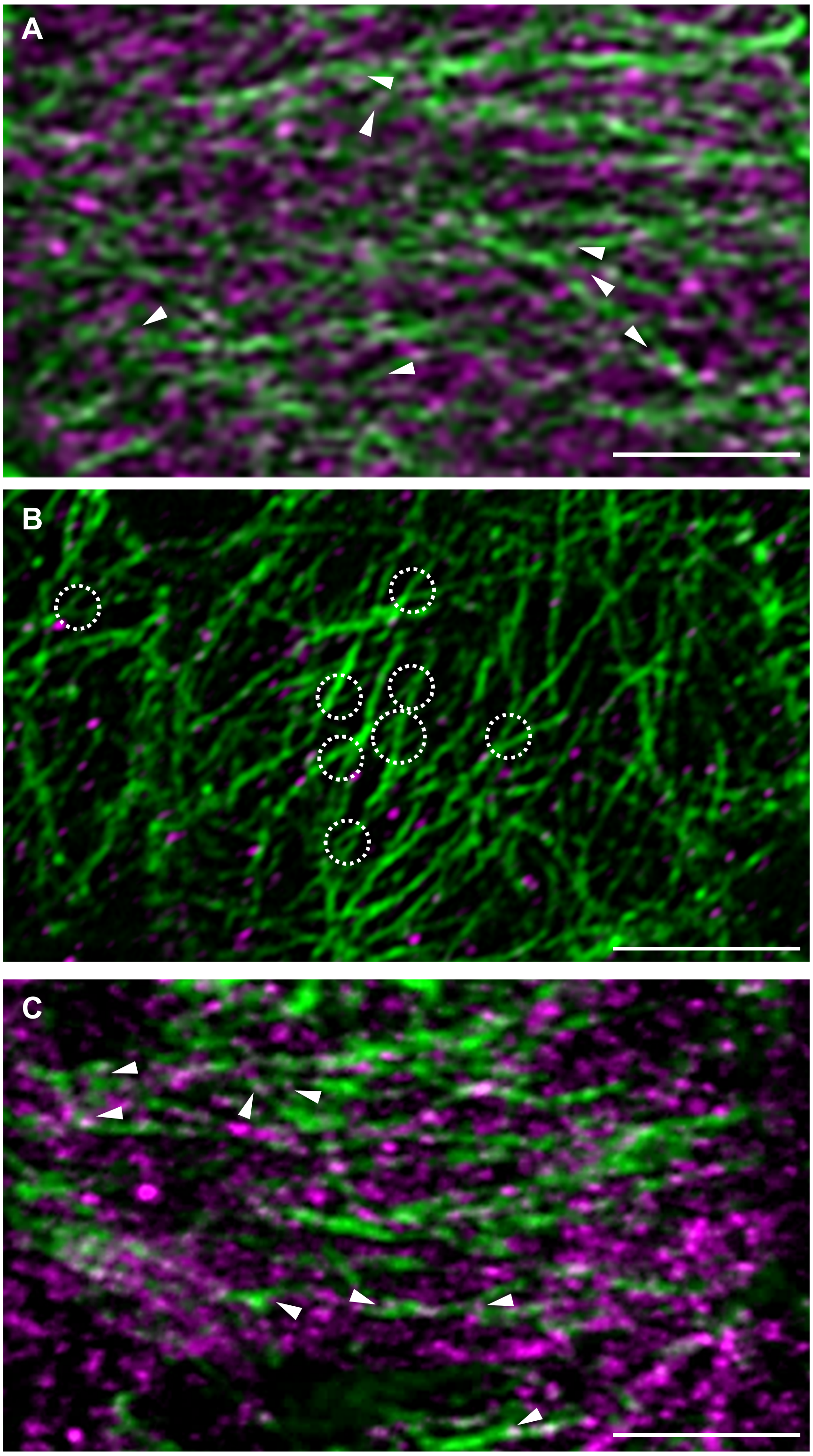


**Supplementary Fig. S3 Association of SIMK with branching points at cortical microtubules in alfalfa control and transgenic lines.** Representative merged images showing association of SIMK (magenta; arrowheads) with cortical microtubules (green) at branching points in control RSY (**A**) and transgenic GFP-SIMK (**C**) lines. Representative merged image showing missing SIMK at branching points (circles) of cortical microtubules in the transgenic SIMKKi line (**B**). SIMK in the GFP-SIMK line (**C**) is pseudocolored in magenta and microtubules in green for consistency with the other images. Scale bar = 5 μm (A-C).

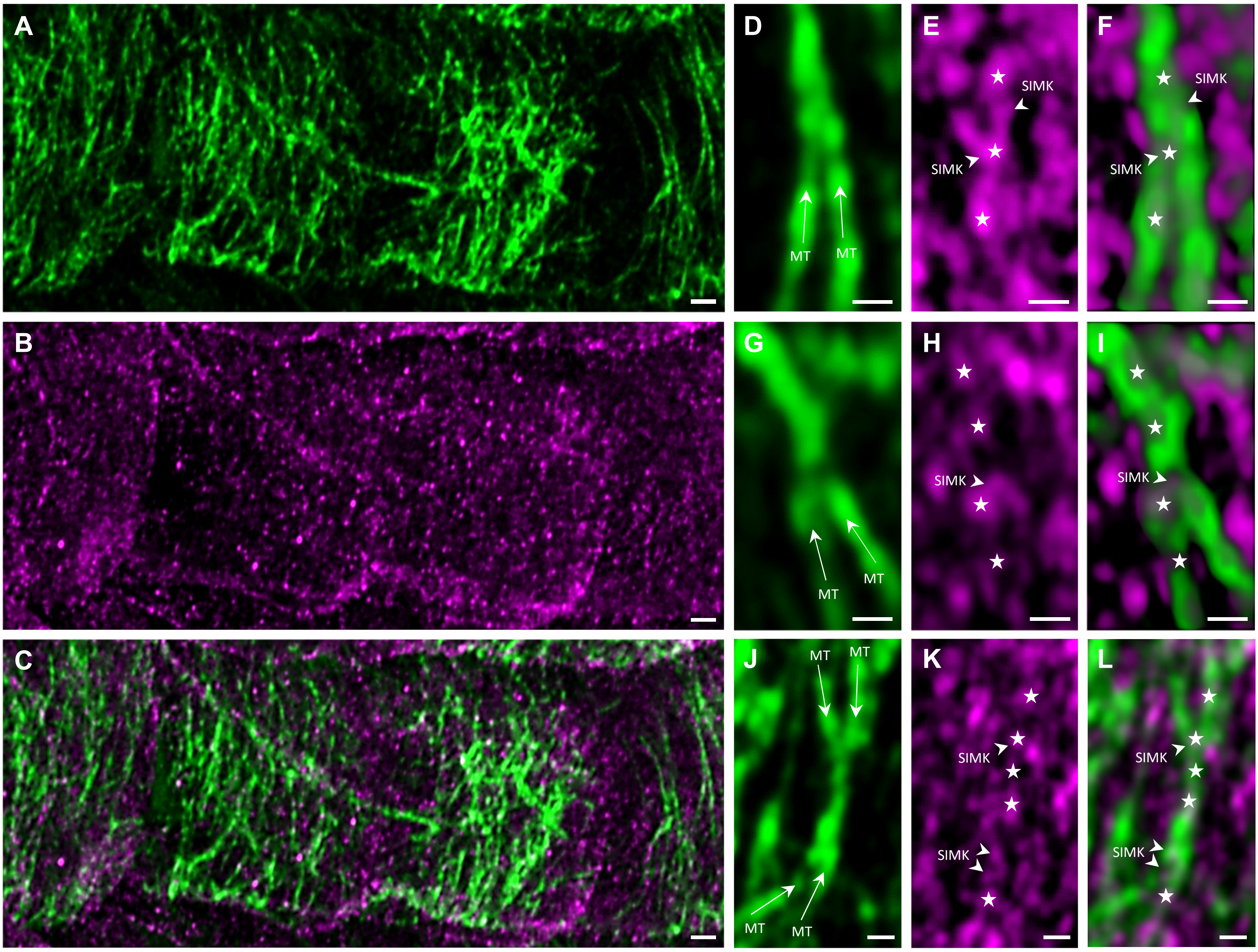


**Supplementary Fig. S4 SIMK association with bundling and branching points at cortical microtubules in alfalfa GFP-SIMK transgenic line.** Overview of the cortical layer of root epidermal cells showing cortical microtubules (green), SIMK (magenta) and merged image (**A-C**). Details depicting events of microtubule bundling or branching with related localization pattern of SIMK (**D-L**). Individual images of cortical microtubules (green; arrows in **D, G, J**) and certain SIMK fraction (magenta; **E, H, K**) in merged images (**F, I, L**) showing spot-like association of SIMK with bundled segments (asterisks) and branching points (arrowheads) of cortical microtubules. Note that in addition of SIMK associated with microtubule bundling/branching points, it is located also in cortical cytoplasm unrelated to cortical microtubules (**E, H, K, F, I, L**). Only SIMK associated with microtubule bundling/branching points is marked by asterisks and arrowheads. SIMK in the GFP-SIMK line is pseudocoloured in magenta and microtubules in green for consistency with the other images. Images are presented as Maximum intensity projections (**A-E, G-H, J-L**) or 3-D rendering using transparency rendering mode (**F, I**) from four consecutive optical sections. Scale bar = 1 µm (A-C), 500 nm (D-L).

**Table S1** Results of two-tailed *F*-test determining significant differences in angle dispersion between two groups

**tagRFP-TUA6 vs SIMKKi tagRFP-TUA6**

Two-tailed *F*-test for variance

| | *Sample 1* | *Sample 2* |
|---|---|---|
| Mean | 96.33950617 | 90.66115702 |
| Variance | 299.1573116 | 16.70922865 |
| Number of Observations | 162 | 121 |
| Degrees of Freedom | 161 | 120 |
| F Statistic | 17.90371763 | |
| P(F<=f) | 8.9296E-46 | |
| F Critical Value | 1.329597385 | |

**tagRFP-TUA6 vs GFP-SIMKtagRFP-TUA6**

Two-tailed *F*-test for variance

| | *Sample 1* | *Sample 2* |
|---|---|---|
| Mean | 91.05517241 | 90.66115702 |
| Variance | 12.60804598 | 16.70922865 |
| Number of Observations | 145 | 121 |
| Degrees of Freedom | 144 | 120 |
| F Statistic | 0.754555835 | |
| P(F<=f) | 0.05287582 | |
| F Critical Value | 0.750974018 | |

**SIMKKitagRFP-TUA6 vs GFP-SIMKtagRFP-TUA6**

Two-tailed *F*-test for variance

| | *Sample 1* | *Sample 2* |
|---|---|---|
| Mean | 96.33950617 | 91.05517241 |
| Variance | 299.1573116 | 12.60804598 |
| Number of Observations | 162 | 145 |
| Degrees of Freedom | 161 | 144 |
| F Statistic | 23.72749212 | |
| P(F<=f) | 2.58854E-61 | |
| F Critical Value | 1.308396518 | |

**RSY vs SIMKKi**

Two-tailed *F*-test for variance

| | *Sample 1* | *Sample 2* |
|---|---|---|
| Mean | 89.41697 | 90.3768538 |
| Variance | 32.81304 | 73.0270811 |
| Number of Observations | 529 | 491 |
| Degrees of Freedom | 528 | 490 |
| F Statistic | 0.449327 | |
| P(F<=f) | 0 | |
| F Critical Value | 0.864312 | |

**RSY vs GFP-SIMK**

Two-tailed *F*-test for variance

| | *Sample 1* | *Sample 2* |
|---|---|---|
| Mean | 89.41697 | 89.6745805 |
| Variance | 32.81304 | 36.3098496 |
| Number of Observations | 529 | 626 |
| Degrees of Freedom | 528 | 625 |
| F Statistic | 0.903695 | |
| P(F<=f) | 0.113806 | |
| F Critical Value | 0.871033 | |

**SIMKKi vs GFP-SIMK**

Two-tailed *F*-test for variance

| | *Sample 1* | *Sample 2* |
|---|---|---|
| Mean | 90.37685 | 89.6745805 |
| Variance | 73.02708 | 36.3098496 |
| Number of Observations | 491 | 626 |
| Degrees of Freedom | 490 | 625 |
| F Statistic | 2.01122 | |
| P(F<=f) (1) | 1.01E-16 | |
| F Critical Value | 1.150032 | |
